# Overexpression of CCK Neuropeptide in brain is linked to ZBTB20 mutations: potential diagnostic relevance in glioblastoma

**DOI:** 10.1186/s12920-025-02218-0

**Published:** 2025-10-07

**Authors:** Tikam Chand Dakal, Narendra Kumar Sharma, Vikas Yadav, Abhishek Kumar, Peng Chen, Ingo Schmidt-Wolf, Jarek Maciaczyk, Amit Sharma

**Affiliations:** 1 Genome and Computational Biology Lab, Department of Biotechnology, Mohanlal Sukhadia University, Udaipur, Rajasthan 313001 India; 2 Department of Bioscience and Biotechnology, Banasthali Vidyapith, Tonk, Rajasthan 304022 India; 3 School of Life Sciences, Jawaharlal Nehru University, New Delhi, 110067 India; 4 Institute of Bioinformatics, International Technology Park, Bangalore, 560066 India; 5 Manipal Academy of Higher Education (MAHE), Manipal, Karnataka 576104 India; 6 Department of Integrated Oncology, Center for Integrated Oncology (CIO), University Hospital of Bonn, Bonn, 53127 Germany; 7 Department of Stereotactic and Functional Neurosurgery, University Hospital of Bonn, Bonn, 53127 Germany

**Keywords:** Glioblastoma, CCK gene expression, IDH1 mutations, Brain cortex, Brain frontal cortex

## Abstract

Glioblastoma (GBM), the most malignant central nervous system cancer, has a median survival rate of 14–16 months. GBM patients have a poor prognosis despite rigorous multi-modal treatments like surgical resection, chemotherapy, and radiation. A systematic bioinformatics analysis of GXA gene expression datasets showed thirty-three overexpressed genes in GBM that were enriched in neuroactive ligand receptor interaction. Subsequently, bulk brain tissue gene expression profiling showed high to moderate expression of CCK, VGF, APLN, CNR1, GRIA4, and PDYN in the cortex, frontal cortex-BA9, BA24 region, cerebellar hemisphere, cerebellum, hippocampus, amygdala, basal ganglia, hypothalamus, substantia nigra, and spinal cord. Interesting, the cortex, frontal cortex-BA9, and BA24 regions had unbelievably high CCK expression. Brain cortex and frontal lobe mutational scoring showed significant rates of IDH1 (49%), TERT (25%), SETD2 (17%), and BRAF (9%). We initially confirmed glioblastoma (GBM) malignancy and therapeutic responses to CCK overexpression and IDH1 mutations in cerebral cortex and frontal cortex tissue samples. However, Mann–Whitney analysis revealed ZBTB20 mutations cause CCK expression, not IDH1 mutations. Overall, CCK expression and ZBTB20 mutations may be used in therapeutic treatments as predictive and diagnostic markers. The expression of CCK was not linked with overall survival and excludes its potential application as prognostic biomarker for GBM. This study showed that CCK and ZBTB20 based predictive and diagnostic technologies can improve precision and personalization of targeted cancer therapies for GBM patients. Nevertheless, experimental evidences have yet to reveal additional therapeutically meaningful connections between CCK expression and ZBTB20 mutations.

**Supplementary Information:**

The online version contains supplementary material available at 10.1186/s12920-025-02218-0.

## Introduction

Gliomas, which exhibit a spectrum of benign and malignant characteristics, are characterised by discernible genetic variations and associated clinical implications. The classification of infiltrating lesions by the World Health Organisation (WHO) involves categorizing them into three groups: low-grade gliomas (LGG), and high-grade gliomas (HGG) and glioblastoma. This classification is based on histopathological features that have been developed from the Bailey and Cushing classification system [1]. The rapid proliferation of integrated, multi-platform genomic sequencing has transformed our understanding of gliomas, but molecular subtyping has helped reveal LGG and GBM subtypes over the past decade [2–4].

The expression of the cholecystokinin (CCK) receptor has been observed in several tumour types due to the capacity of CCK to facilitate the survival and proliferation of neoplastic cells. The CCK gene is responsible for encoding a protein that belongs to the gastrin/cholecystokinin protein family. The preproprotein undergoes proteolytic processing, resulting in the production of several protein products, such as cholecystokinin-8, −12, −33, and other peptide hormones. Previous studies have demonstrated that the encoded peptides possess the ability to modulate both stomach acid secretion and food intake. The neuronal activity in the brain may be influenced by a sulfated variant of cholecystokinin-8. The process of alternative splicing leads to the generation of numerous transcript variants.

IDH1/2, ATRX, BRAF, TERT, and H3K27M have been found to play important roles [5]. In fact, the 2016 WHO classification of tumours recommends molecular technologies to diagnose gliomas, noting enhanced marker accuracy over histological methods [1]. These mutations also cause LGG to become HGG by paired genomic sequencing [6]. These molecular techniques have transformed glioma pathogenesis, tumour categorization, patient therapy, and novel therapeutic approaches.

## Materials and methods

### Retrieval of genes differentially overexpressed in the glioblastoma

Genes differentially overexpressed in the Glioblastoma were retrieved from the public database (https://www.ebi.ac.uk/gxa/) (Accessed on July 28, 2023; Time: 14:30PM IST). From the list of the genes differentially upregulated and downregulated (based on their log_2_-fold change value and p-value ≤ 0.01) (Supplemental Table 1), only genes upregulated were downloaded and saved into the local computer for further gene enrichment and pathway analysis (Table 1).

**Table 1:** List of upregulated genes in Glioblastoma with their description (Please remove empty columns)

ENSEMBL_GENE_ID	Gene name	Related genes	Species
ENSG00000001561	ectonucleotide pyrophosphatase/phosphodiesterase 4(ENPP4)	RG	Homo sapiens
ENSG00000001626	CF transmembrane conductance regulator (CFTR)	RG	Homo sapiens
ENSG00000002587	heparan sulfate-glucosamine 3-sulfotransferase 1 (HS3ST1)	RG	Homo sapiens
ENSG00000002726	amine oxidase copper containing 1 (AOC1)	RG	Homo sapiens
ENSG00000002933	transmembrane protein 176 A (TMEM176A)	RG	Homo sapiens
ENSG00000003989	solute carrier family 7 member 2 (SLC7A2)	RG	Homo sapiens
ENSG00000004799	pyruvate dehydrogenase kinase 4 (PDK4)	RG	Homo sapiens
ENSG00000005001	serine protease 22 (PRSS22)	RG	Homo sapiens
ENSG00000005381	myeloperoxidase (MPO)	RG	Homo sapiens
ENSG00000005471	ATP binding cassette subfamily B member 4 (ABCB4)	RG	Homo sapiens
ENSG00000006210	C-X3-C motif chemokine ligand 1 (CX3CL1)	RG	Homo sapiens
ENSG00000006432	mitogen-activated protein kinase kinase kinase 9 (MAP3K9)	RG	Homo sapiens
ENSG00000006555	tetratricopeptide repeat domain 22 (TTC22)	RG	Homo sapiens
ENSG00000007062	prominin 1 (PROM1)	RG	Homo sapiens
ENSG00000008196	transcription factor AP-2 beta (TFAP2B)	RG	Homo sapiens
ENSG00000010319	semaphorin 3G (SEMA3G)	RG	Homo sapiens
ENSG00000016082	ISL LIM homeobox 1 (ISL1)	RG	Homo sapiens
ENSG00000019169	macrophage receptor with collagenous structure (MARCO)	RG	Homo sapiens
ENSG00000019505	synaptotagmin 13 (SYT13)	RG	Homo sapiens
ENSG00000019582	CD74 molecule (CD74)	RG	Homo sapiens
ENSG00000021355	serpin family B member 1 (SERPINB1)	RG	Homo sapiens
ENSG00000040731	cadherin 10 (CDH10)	RG	Homo sapiens
ENSG00000041515	myosin XVI (MYO16)	RG	Homo sapiens
ENSG00000046604	desmoglein 2 (DSG2)	RG	Homo sapiens
ENSG00000046653	glycoprotein M6B (GPM6B)	RG	Homo sapiens
ENSG00000046774	MAGE family member C2 (MAGEC2)	RG	Homo sapiens
ENSG00000046889	phosphatidylinositol-3,4,5-trisphosphate dependent Rac exchange factor 2 (PREX2)	RG	Homo sapiens
ENSG00000047365	ArfGAP with RhoGAP domain, ankyrin repeat and PH domain 2 (ARAP2)	RG	Homo sapiens
ENSG00000048740	CUGBP Elav-like family member 2 (CELF2)	RG	Homo sapiens
ENSG00000050327	Rho guanine nucleotide exchange factor 5 (ARHGEF5)	RG	Homo sapiens
ENSG00000054219	lymphocyte antigen 75 (LY75)	RG	Homo sapiens
ENSG00000060718	collagen type XI alpha 1 chain (COL11A1)	RG	Homo sapiens
ENSG00000063515	goosecoid homeobox 2 (GSC2)	RG	Homo sapiens
ENSG00000064195	distal-less homeobox 3 (DLX3)	RG	Homo sapiens
ENSG00000065320	netrin 1 (NTN1)	RG	Homo sapiens
ENSG00000065325	glucagon like peptide 2 receptor (GLP2R)	RG	Homo sapiens
ENSG00000065609	synaptosome associated protein 91 (SNAP91)	RG	Homo sapiens
ENSG00000065833	malic enzyme 1 (ME1)	RG	Homo sapiens
ENSG00000067715	synaptotagmin 1 (SYT1)	RG	Homo sapiens
ENSG00000068781	STON1-GTF2A1L readthrough (STON1-GTF2A1L)	RG	Homo sapiens
ENSG00000069122	adhesion G protein-coupled receptor F5 (ADGRF5)	RG	Homo sapiens
ENSG00000069482	galanin and GMAP prepropeptide (GAL)	RG	Homo sapiens
ENSG00000073756	prostaglandin-endoperoxide synthase 2 (PTGS2)	RG	Homo sapiens
ENSG00000074211	protein phosphatase 2 regulatory subunit Bgamma (PPP2R2C)	RG	Homo sapiens
ENSG00000074966	TXK tyrosine kinase (TXK)	RG	Homo sapiens
ENSG00000075891	paired box 2 (PAX2)	RG	Homo sapiens
ENSG00000076826	calmodulin regulated spectrin associated protein family member 3 (CAMSAP3)	RG	Homo sapiens
ENSG00000076944	syntaxin binding protein 2 (STXBP2)	RG	Homo sapiens
ENSG00000077009	nicotinamide riboside kinase 2 (NMRK2)	RG	Homo sapiens
ENSG00000077063	cortactin binding protein 2 (CTTNBP2)	RG	Homo sapiens
ENSG00000077238	interleukin 4 receptor (IL4R)	RG	Homo sapiens
ENSG00000078081	lysosomal associated membrane protein 3 (LAMP3)	RG	Homo sapiens
ENSG00000081052	collagen type IV alpha 4 chain (COL4A4)	RG	Homo sapiens
ENSG00000082175	progesterone receptor (PGR)	RG	Homo sapiens
ENSG00000082497	SERTA domain containing 4 (SERTAD4)	RG	Homo sapiens
ENSG00000083782	epiphycan (EPYC)	RG	Homo sapiens
ENSG00000085563	ATP binding cassette subfamily B member 1 (ABCB1)	RG	Homo sapiens
ENSG00000088386	solute carrier family 15 member 1 (SLC15A1)	RG	Homo sapiens
ENSG00000088882	carboxypeptidase X, M14 family member 1 (CPXM1)	RG	Homo sapiens
ENSG00000089116	LIM homeobox 5 (LHX5)	RG	Homo sapiens
ENSG00000089327	FXYD domain containing ion transport regulator 5 (FXYD5)	RG	Homo sapiens
ENSG00000090376	interleukin 1 receptor associated kinase 3 (IRAK3)	RG	Homo sapiens
ENSG00000091129	neuronal cell adhesion molecule (NRCAM)	RG	Homo sapiens
ENSG00000091972	CD200 molecule (CD200)	RG	Homo sapiens
ENSG00000092421	semaphorin 6 A (SEMA6A)	RG	Homo sapiens
ENSG00000095752	interleukin 11 (IL11)	RG	Homo sapiens
ENSG00000096696	desmoplakin (DSP)	RG	Homo sapiens
ENSG00000099998	gamma-glutamyltransferase 5 (GGT5)	RG	Homo sapiens
ENSG00000100060	MFNG O-fucosylpeptide 3-beta-N-acetylglucosaminyltransferase (MFNG)	RG	Homo sapiens
ENSG00000100285	neurofilament heavy chain (NEFH)	RG	Homo sapiens
ENSG00000100290	BCL2 interacting killer (BIK)	RG	Homo sapiens
ENSG00000100433	potassium two pore domain channel subfamily K member 10 (KCNK10)	RG	Homo sapiens
ENSG00000100473	cochlin (COCH)	RG	Homo sapiens
ENSG00000100558	pleckstrin 2 (PLEK2)	RG	Homo sapiens
ENSG00000100739	bradykinin receptor B1 (BDKRB1)	RG	Homo sapiens
ENSG00000101134	docking protein 5 (DOK5)	RG	Homo sapiens
ENSG00000101188	neurotensin receptor 1 (NTSR1)	RG	Homo sapiens
ENSG00000101204	cholinergic receptor nicotinic alpha 4 subunit (CHRNA4)	RG	Homo sapiens
ENSG00000101327	prodynorphin (PDYN)	RG	Homo sapiens
ENSG00000101336	HCK proto-oncogene, Src family tyrosine kinase (HCK)	RG	Homo sapiens
ENSG00000101445	protein phosphatase 1 regulatory subunit 16B (PPP1R16B)	RG	Homo sapiens
ENSG00000102230	phosphate cytidylyltransferase 1B, choline (PCYT1B)	RG	Homo sapiens
ENSG00000102287	gamma-aminobutyric acid type A receptor subunit epsilon (GABRE)	RG	Homo sapiens
ENSG00000102678	fibroblast growth factor 9 (FGF9)	RG	Homo sapiens
ENSG00000102755	fms related receptor tyrosine kinase 1 (FLT1)	RG	Homo sapiens
ENSG00000103241	forkhead box F1 (FOXF1)	RG	Homo sapiens
ENSG00000103460	TOX high mobility group box family member 3 (TOX3)	RG	Homo sapiens
ENSG00000103811	cathepsin H (CTSH)	RG	Homo sapiens
ENSG00000104213	platelet derived growth factor receptor like (PDGFRL)	RG	Homo sapiens
ENSG00000104368	plasminogen activator, tissue type (PLAT)	RG	Homo sapiens
ENSG00000104413	epithelial splicing regulatory protein 1 (ESRP1)	RG	Homo sapiens
ENSG00000105278	zinc finger RNA binding protein 2 (ZFR2)	RG	Homo sapiens
ENSG00000105509	hyaluronan synthase 1 (HAS1)	RG	Homo sapiens
ENSG00000105976	MET proto-oncogene, receptor tyrosine kinase (MET)	RG	Homo sapiens
ENSG00000106123	EPH receptor B6 (EPHB6)	RG	Homo sapiens
ENSG00000106278	protein tyrosine phosphatase receptor type Z1(PTPRZ1)	RG	Homo sapiens
ENSG00000106511	mesenchyme homeobox 2(MEOX2)	RG	Homo sapiens
ENSG00000106565	transmembrane protein 176B(TMEM176B)	RG	Homo sapiens
ENSG00000106852	LIM homeobox 6(LHX6)	RG	Homo sapiens
ENSG00000107159	carbonic anhydrase 9(CA9)	RG	Homo sapiens
ENSG00000108342	colony stimulating factor 3(CSF3)	RG	Homo sapiens
ENSG00000108691	C–C motif chemokine ligand 2(CCL2)	RG	Homo sapiens
ENSG00000108924	HLF transcription factor, PAR bZIP family member(HLF)	RG	Homo sapiens
ENSG00000109265	capping protein inhibiting regulator of actin dynamics(CRACD)	RG	Homo sapiens
ENSG00000110680	calcitonin related polypeptide alpha(CALCA)	RG	Homo sapiens
ENSG00000110852	C-type lectin domain family 2 member B(CLEC2B)	RG	Homo sapiens
ENSG00000111058	acyl-CoA synthetase short chain family member 3(ACSS3)	RG	Homo sapiens
ENSG00000111199	transient receptor potential cation channel subfamily V member 4(TRPV4)	RG	Homo sapiens
ENSG00000111262	potassium voltage-gated channel subfamily A member 1(KCNA1)	RG	Homo sapiens
ENSG00000111424	vitamin D receptor(VDR)	RG	Homo sapiens
ENSG00000111799	collagen type XII alpha 1 chain(COL12A1)	RG	Homo sapiens
ENSG00000111846	glucosaminyl (N-acetyl) transferase 2 (I blood group)(GCNT2)	RG	Homo sapiens
ENSG00000111913	RHO family interacting cell polarization regulator 2(RIPOR2)	RG	Homo sapiens
ENSG00000112333	nuclear receptor subfamily 2 group E member 1(NR2E1)	RG	Homo sapiens
ENSG00000112379	ARFGEF family member 3(ARFGEF3)	RG	Homo sapiens
ENSG00000112562	SPARC related modular calcium binding 2(SMOC2)	RG	Homo sapiens
ENSG00000112796	ectonucleotide pyrophosphatase/phosphodiesterase family member 5(ENPP5)	RG	Homo sapiens
ENSG00000112812	serine protease 16(PRSS16)	RG	Homo sapiens
ENSG00000113430	iroquois homeobox 4(IRX4)	RG	Homo sapiens
ENSG00000113749	histamine receptor H2(HRH2)	RG	Homo sapiens
ENSG00000114790	Rho guanine nucleotide exchange factor 26(ARHGEF26)	RG	Homo sapiens
ENSG00000114805	phospholipase C eta 1(PLCH1)	RG	Homo sapiens
ENSG00000115112	transcription factor CP2 like 1(TFCP2L1)	RG	Homo sapiens
ENSG00000115290	growth factor receptor bound protein 14(GRB14)	RG	Homo sapiens
ENSG00000115457	insulin like growth factor binding protein 2(IGFBP2)	RG	Homo sapiens
ENSG00000116544	DLG associated protein 3(DLGAP3)	RG	Homo sapiens
ENSG00000116745	retinoid isomerohydrolase RPE65(RPE65)	RG	Homo sapiens
ENSG00000117598	phospholipid phosphatase related 5(PLPPR5)	RG	Homo sapiens
ENSG00000117707	prospero homeobox 1(PROX1)	RG	Homo sapiens
ENSG00000118432	cannabinoid receptor 1(CNR1)	RG	Homo sapiens
ENSG00000118946	protocadherin 17(PCDH17)	RG	Homo sapiens
ENSG00000118971	cyclin D2(CCND2)	RG	Homo sapiens
ENSG00000119125	guanine deaminase(GDA)	RG	Homo sapiens
ENSG00000119698	protein phosphatase 4 regulatory subunit 4(PPP4R4)	RG	Homo sapiens
ENSG00000119714	G protein-coupled receptor 68(GPR68)	RG	Homo sapiens
ENSG00000119866	BCL11 transcription factor A(BCL11A)	RG	Homo sapiens
ENSG00000120093	homeobox B3(HOXB3)	RG	Homo sapiens
ENSG00000120149	msh homeobox 2(MSX2)	RG	Homo sapiens
ENSG00000120278	pleckstrin homology and RhoGEF domain containing G1(PLEKHG1)	RG	Homo sapiens
ENSG00000120279	MYC target 1(MYCT1)	RG	Homo sapiens
ENSG00000121207	lecithin retinol acyltransferase(LRAT)	RG	Homo sapiens
ENSG00000121316	phospholipase B domain containing 1(PLBD1)	RG	Homo sapiens
ENSG00000121361	potassium inwardly rectifying channel subfamily J member 8(KCNJ8)	RG	Homo sapiens
ENSG00000121742	gap junction protein beta 6(GJB6)	RG	Homo sapiens
ENSG00000121898	carboxypeptidase X, M14 family member 2(CPXM2)	RG	Homo sapiens
ENSG00000122025	fms related receptor tyrosine kinase 3(FLT3)	RG	Homo sapiens
ENSG00000122641	inhibin subunit beta A(INHBA)	RG	Homo sapiens
ENSG00000122733	PHD finger protein 24(PHF24)	RG	Homo sapiens
ENSG00000123080	cyclin dependent kinase inhibitor 2C(CDKN2C)	RG	Homo sapiens
ENSG00000123700	potassium inwardly rectifying channel subfamily J member 2(KCNJ2)	RG	Homo sapiens
ENSG00000124102	peptidase inhibitor 3(PI3)	RG	Homo sapiens
ENSG00000124103	family with sequence similarity 209 member A(FAM209A)	RG	Homo sapiens
ENSG00000124107	secretory leukocyte peptidase inhibitor(SLPI)	RG	Homo sapiens
ENSG00000124126	phosphatidylinositol-3,4,5-trisphosphate dependent Rac exchange factor 1(PREX1)	RG	Homo sapiens
ENSG00000124256	Z-DNA binding protein 1(ZBP1)	RG	Homo sapiens
ENSG00000124490	cysteine rich secretory protein 2(CRISP2)	RG	Homo sapiens
ENSG00000124882	epiregulin(EREG)	RG	Homo sapiens
ENSG00000125378	bone morphogenetic protein 4(BMP4)	RG	Homo sapiens
ENSG00000125384	prostaglandin E receptor 2(PTGER2)	RG	Homo sapiens
ENSG00000125730	complement C3(C3)	RG	Homo sapiens
ENSG00000125850	ovo like zinc finger 2(OVOL2)	RG	Homo sapiens
ENSG00000125869	lysosomal associated membrane protein family member 5(LAMP5)	RG	Homo sapiens
ENSG00000125878	transcription factor 15(TCF15)	RG	Homo sapiens
ENSG00000126010	gastrin releasing peptide receptor(GRPR)	RG	Homo sapiens
ENSG00000127074	regulator of G protein signaling 13(RGS13)	RG	Homo sapiens
ENSG00000127955	G protein subunit alpha i1(GNAI1)	RG	Homo sapiens
ENSG00000128165	adrenomedullin 2(ADM2)	RG	Homo sapiens
ENSG00000128422	keratin 17(KRT17)	RG	Homo sapiens
ENSG00000128564	VGF nerve growth factor inducible(VGF)	RG	Homo sapiens
ENSG00000128610	FEZ family zinc finger 1(FEZF1)	RG	Homo sapiens
ENSG00000128917	delta like canonical Notch ligand 4(DLL4)	RG	Homo sapiens
ENSG00000129151	gamma-butyrobetaine hydroxylase 1(BBOX1)	RG	Homo sapiens
ENSG00000129450	sialic acid binding Ig like lectin 9(SIGLEC9)	RG	Homo sapiens
ENSG00000129993	CBFA2/RUNX1 partner transcriptional co-repressor 3(CBFA2T3)	RG	Homo sapiens
ENSG00000130222	growth arrest and DNA damage inducible gamma(GADD45G)	RG	Homo sapiens
ENSG00000130294	kinesin family member 1A(KIF1A)	RG	Homo sapiens
ENSG00000130487	kelch domain containing 7B(KLHDC7B)	RG	Homo sapiens
ENSG00000130700	GATA binding protein 5(GATA5)	RG	Homo sapiens
ENSG00000130762	Rho guanine nucleotide exchange factor 16(ARHGEF16)	RG	Homo sapiens
ENSG00000131203	indoleamine 2,3-dioxygenase 1(IDO1)	RG	Homo sapiens
ENSG00000131737	keratin 34(KRT34)	RG	Homo sapiens
ENSG00000131831	retinoic acid induced 2(RAI2)	RG	Homo sapiens
ENSG00000132530	XIAP associated factor 1(XAF1)	RG	Homo sapiens
ENSG00000132688	nestin(NES)	RG	Homo sapiens
ENSG00000132911	neuromedin U receptor 2(NMUR2)	RG	Homo sapiens
ENSG00000133026	myosin heavy chain 10(MYH10)	RG	Homo sapiens
ENSG00000133101	cyclin A1(CCNA1)	RG	Homo sapiens
ENSG00000133110	periostin(POSTN)	RG	Homo sapiens
ENSG00000134121	cell adhesion molecule L1 like(CHL1)	RG	Homo sapiens
ENSG00000134202	glutathione S-transferase mu 3(GSTM3)	RG	Homo sapiens
ENSG00000134602	serine/threonine kinase 26(STK26)	RG	Homo sapiens
ENSG00000134765	desmocollin 1(DSC1)	RG	Homo sapiens
ENSG00000135063	endosomal transmembrane epsin interactor 1(ENTREP1)	RG	Homo sapiens
ENSG00000135097	musashi RNA binding protein 1(MSI1)	RG	Homo sapiens
ENSG00000135127	BICD family like cargo adaptor 1(BICDL1)	RG	Homo sapiens
ENSG00000135318	5'-nucleotidase ecto(NT5E)	RG	Homo sapiens
ENSG00000135373	ETS homologous factor(EHF)	RG	Homo sapiens
ENSG00000135424	integrin subunit alpha 7(ITGA7)	RG	Homo sapiens
ENSG00000135447	protein phosphatase 1 regulatory inhibitor subunit 1A(PPP1R1A)	RG	Homo sapiens
ENSG00000135625	early growth response 4(EGR4)	RG	Homo sapiens
ENSG00000135750	potassium two pore domain channel subfamily K member 1(KCNK1)	RG	Homo sapiens
ENSG00000135929	cytochrome P450 family 27 subfamily A member 1(CYP27A1)	RG	Homo sapiens
ENSG00000136002	Rho guanine nucleotide exchange factor 4(ARHGEF4)	RG	Homo sapiens
ENSG00000136327	NK2 homeobox 8(NKX2-8)	RG	Homo sapiens
ENSG00000136352	NK2 homeobox 1(NKX2-1)	RG	Homo sapiens
ENSG00000136574	GATA binding protein 4(GATA4)	RG	Homo sapiens
ENSG00000136695	interleukin 36 receptor antagonist(IL36RN)	RG	Homo sapiens
ENSG00000136842	tropomodulin 1(TMOD1)	RG	Homo sapiens
ENSG00000136960	ectonucleotide pyrophosphatase/phosphodiesterase 2(ENPP2)	RG	Homo sapiens
ENSG00000137090	doublesex and mab-3 related transcription factor 1(DMRT1)	RG	Homo sapiens
ENSG00000137265	interferon regulatory factor 4(IRF4)	RG	Homo sapiens
ENSG00000137642	sortilin related receptor 1(SORL1)	RG	Homo sapiens
ENSG00000138379	myostatin(MSTN)	RG	Homo sapiens
ENSG00000138449	solute carrier family 40 member 1(SLC40A1)	RG	Homo sapiens
ENSG00000138622	hyperpolarization activated cyclic nucleotide gated potassium channel 4(HCN4)	RG	Homo sapiens
ENSG00000138670	RasGEF domain family member 1B(RASGEF1B)	RG	Homo sapiens
ENSG00000139219	collagen type II alpha 1 chain(COL2A1)	RG	Homo sapiens
ENSG00000139515	pancreatic and duodenal homeobox 1(PDX1)	RG	Homo sapiens
ENSG00000139610	chymotrypsin like elastase 1(CELA1)	RG	Homo sapiens
ENSG00000139910	NOVA alternative splicing regulator 1(NOVA1)	RG	Homo sapiens
ENSG00000139970	reticulon 1(RTN1)	RG	Homo sapiens
ENSG00000140557	ST8 alpha-N-acetyl-neuraminide alpha-2,8-sialyltransferase 2(ST8SIA2)	RG	Homo sapiens
ENSG00000140937	cadherin 11(CDH11)	RG	Homo sapiens
ENSG00000141316	sperm acrosome associated 3(SPACA3)	RG	Homo sapiens
ENSG00000141431	ASXL transcriptional regulator 3(ASXL3)	RG	Homo sapiens
ENSG00000142449	fibrillin 3(FBN3)	RG	Homo sapiens
ENSG00000143469	synaptotagmin 14(SYT14)	RG	Homo sapiens
ENSG00000143674	mitogen-activated protein kinase kinase kinase 21(MAP3K21)	RG	Homo sapiens
ENSG00000143954	regenerating family member 3 gamma(REG3G)	RG	Homo sapiens
ENSG00000144119	complement C1q like 2(C1QL2)	RG	Homo sapiens
ENSG00000144331	zinc finger protein 385B(ZNF385B)	RG	Homo sapiens
ENSG00000144488	espin like(ESPNL)	RG	Homo sapiens
ENSG00000144596	glutamate receptor interacting protein 2(GRIP2)	RG	Homo sapiens
ENSG00000144668	integrin subunit alpha 9(ITGA9)	RG	Homo sapiens
ENSG00000144681	SH3 and cysteine rich domain(STAC)	RG	Homo sapiens
ENSG00000145390	ubiquitin specific peptidase 53(USP53)	RG	Homo sapiens
ENSG00000145423	secreted frizzled related protein 2(SFRP2)	RG	Homo sapiens
ENSG00000145703	IQ motif containing GTPase activating protein 2(IQGAP2)	RG	Homo sapiens
ENSG00000145808	ADAM metallopeptidase with thrombospondin type 1 motif 19(ADAMTS19)	RG	Homo sapiens
ENSG00000145934	teneurin transmembrane protein 2(TENM2)	RG	Homo sapiens
ENSG00000146197	signal peptide, CUB domain and EGF like domain containing 3(SCUBE3)	RG	Homo sapiens
ENSG00000146250	serine protease 35(PRSS35)	RG	Homo sapiens
ENSG00000146469	vasoactive intestinal peptide(VIP)	RG	Homo sapiens
ENSG00000146530	von Willebrand factor D and EGF domains(VWDE)	RG	Homo sapiens
ENSG00000146555	sidekick cell adhesion molecule 1(SDK1)	RG	Homo sapiens
ENSG00000146938	neuroligin 4 X-linked(NLGN4X)	RG	Homo sapiens
ENSG00000147041	synaptotagmin like 5(SYTL5)	RG	Homo sapiens
ENSG00000147588	peripheral myelin protein 2(PMP2)	RG	Homo sapiens
ENSG00000148053	neurotrophic receptor tyrosine kinase 2(NTRK2)	RG	Homo sapiens
ENSG00000148408	calcium voltage-gated channel subunit alpha1 B(CACNA1B)	RG	Homo sapiens
ENSG00000149256	teneurin transmembrane protein 4(TENM4)	RG	Homo sapiens
ENSG00000149573	myelin protein zero like 2(MPZL2)	RG	Homo sapiens
ENSG00000149972	contactin 5(CNTN5)	RG	Homo sapiens
ENSG00000150051	mohawk homeobox(MKX)	RG	Homo sapiens
ENSG00000150556	LY6/PLAUR domain containing 6B(LYPD6B)	RG	Homo sapiens
ENSG00000151490	protein tyrosine phosphatase receptor type O(PTPRO)	RG	Homo sapiens
ENSG00000151702	Fli-1 proto-oncogene, ETS transcription factor(FLI1)	RG	Homo sapiens
ENSG00000151834	gamma-aminobutyric acid type A receptor subunit alpha2(GABRA2)	RG	Homo sapiens
ENSG00000152128	transmembrane protein 163(TMEM163)	RG	Homo sapiens
ENSG00000152137	heat shock protein family B (small) member 8(HSPB8)	RG	Homo sapiens
ENSG00000152315	potassium two pore domain channel subfamily K member 13(KCNK13)	RG	Homo sapiens
ENSG00000152578	glutamate ionotropic receptor AMPA type subunit 4(GRIA4)	RG	Homo sapiens
ENSG00000152784	PR/SET domain 8(PRDM8)	RG	Homo sapiens
ENSG00000152932	RAB3C, member RAS oncogene family(RAB3C)	RG	Homo sapiens
ENSG00000153162	bone morphogenetic protein 6(BMP6)	RG	Homo sapiens
ENSG00000153208	MER proto-oncogene, tyrosine kinase(MERTK)	RG	Homo sapiens
ENSG00000153266	FEZ family zinc finger 2(FEZF2)	RG	Homo sapiens
ENSG00000153291	solute carrier family 25 member 27(SLC25A27)	RG	Homo sapiens
ENSG00000153993	semaphorin 3D(SEMA3D)	RG	Homo sapiens
ENSG00000154342	Wnt family member 3A(WNT3A)	RG	Homo sapiens
ENSG00000155495	MAGE family member C1(MAGEC1)	RG	Homo sapiens
ENSG00000155849	engulfment and cell motility 1(ELMO1)	RG	Homo sapiens
ENSG00000155918	retinoic acid early transcript 1L(RAET1L)	RG	Homo sapiens
ENSG00000156466	growth differentiation factor 6(GDF6)	RG	Homo sapiens
ENSG00000156687	unc-5 netrin receptor D(UNC5D)	RG	Homo sapiens
ENSG00000157404	KIT proto-oncogene, receptor tyrosine kinase(KIT)	RG	Homo sapiens
ENSG00000157445	calcium voltage-gated channel auxiliary subunit alpha2delta 3(CACNA2D3)	RG	Homo sapiens
ENSG00000158014	solute carrier family 30 member 2(SLC30A2)	RG	Homo sapiens
ENSG00000158089	polypeptide N-acetylgalactosaminyltransferase 14(GALNT14)	RG	Homo sapiens
ENSG00000158125	xanthine dehydrogenase(XDH)	RG	Homo sapiens
ENSG00000158473	CD1d molecule(CD1D)	RG	Homo sapiens
ENSG00000158528	protein phosphatase 1 regulatory subunit 9A(PPP1R9A)	RG	Homo sapiens
ENSG00000158731	olfactory receptor family 10 subfamily J member 6 pseudogene(OR10J6P)	RG	Homo sapiens
ENSG00000158825	cytidine deaminase(CDA)	RG	Homo sapiens
ENSG00000158966	cache domain containing 1(CACHD1)	RG	Homo sapiens
ENSG00000159166	ladinin 1(LAD1)	RG	Homo sapiens
ENSG00000159217	insulin like growth factor 2 mRNA binding protein 1(IGF2BP1)	RG	Homo sapiens
ENSG00000159251	actin alpha cardiac muscle 1(ACTC1)	RG	Homo sapiens
ENSG00000160191	phosphodiesterase 9A(PDE9A)	RG	Homo sapiens
ENSG00000161249	dermokine(DMKN)	RG	Homo sapiens
ENSG00000162344	fibroblast growth factor 19(FGF19)	RG	Homo sapiens
ENSG00000162367	TAL bHLH transcription factor 1, erythroid differentiation factor(TAL1)	RG	Homo sapiens
ENSG00000162595	DIRAS family GTPase 3(DIRAS3)	RG	Homo sapiens
ENSG00000162624	LIM homeobox 8(LHX8)	RG	Homo sapiens
ENSG00000162670	BMP/retinoic acid inducible neural specific 3(BRINP3)	RG	Homo sapiens
ENSG00000162892	interleukin 24(IL24)	RG	Homo sapiens
ENSG00000162894	Fc mu receptor(FCMR)	RG	Homo sapiens
ENSG00000163216	small proline rich protein 2D(SPRR2D)	RG	Homo sapiens
ENSG00000163235	transforming growth factor alpha(TGFA)	RG	Homo sapiens
ENSG00000163285	gamma-aminobutyric acid type A receptor subunit gamma1(GABRG1)	RG	Homo sapiens
ENSG00000163293	NIPA like domain containing 1(NIPAL1)	RG	Homo sapiens
ENSG00000163421	prokineticin 2(PROK2)	RG	Homo sapiens
ENSG00000163501	Indian hedgehog signaling molecule(IHH)	RG	Homo sapiens
ENSG00000163623	NK6 homeobox 1(NKX6-1)	RG	Homo sapiens
ENSG00000163624	CDP-diacylglycerol synthase 1(CDS1)	RG	Homo sapiens
ENSG00000163710	procollagen C-endopeptidase enhancer 2(PCOLCE2)	RG	Homo sapiens
ENSG00000163739	C-X-C motif chemokine ligand 1(CXCL1)	RG	Homo sapiens
ENSG00000163793	DnaJ heat shock protein family (Hsp40) member C5 gamma(DNAJC5G)	RG	Homo sapiens
ENSG00000164093	paired like homeodomain 2(PITX2)	RG	Homo sapiens
ENSG00000164128	neuropeptide Y receptor Y1(NPY1R)	RG	Homo sapiens
ENSG00000164220	coagulation factor II thrombin receptor like 2(F2RL2)	RG	Homo sapiens
ENSG00000164266	serine peptidase inhibitor Kazal type 1(SPINK1)	RG	Homo sapiens
ENSG00000164283	endothelial cell specific molecule 1(ESM1)	RG	Homo sapiens
ENSG00000164458	T-box transcription factor T(TBXT)	RG	Homo sapiens
ENSG00000164484	transmembrane protein 200A(TMEM200A)	RG	Homo sapiens
ENSG00000164530	peptidase inhibitor 16(PI16)	RG	Homo sapiens
ENSG00000164532	T-box transcription factor 20(TBX20)	RG	Homo sapiens
ENSG00000164626	potassium two pore domain channel subfamily K member 5(KCNK5)	RG	Homo sapiens
ENSG00000164647	STEAP family member 1(STEAP1)	RG	Homo sapiens
ENSG00000164694	fibronectin type III domain containing 1(FNDC1)	RG	Homo sapiens
ENSG00000164761	TNF receptor superfamily member 11b(TNFRSF11B)	RG	Homo sapiens
ENSG00000164841	transmembrane protein 74(TMEM74)	RG	Homo sapiens
ENSG00000164853	UNC homeobox(UNCX)	RG	Homo sapiens
ENSG00000165061	zinc finger matrin-type 4(ZMAT4)	RG	Homo sapiens
ENSG00000165078	carboxypeptidase A6(CPA6)	RG	Homo sapiens
ENSG00000165092	aldehyde dehydrogenase 1 family member A1(ALDH1A1)	RG	Homo sapiens
ENSG00000165349	solute carrier family 7 member 3(SLC7A3)	RG	Homo sapiens
ENSG00000165449	solute carrier family 16 member 9(SLC16A9)	RG	Homo sapiens
ENSG00000165521	EMAP like 5(EML5)	RG	Homo sapiens
ENSG00000165905	LARGE xylosyl- and glucuronyltransferase 2(LARGE2)	RG	Homo sapiens
ENSG00000165929	tandem C2 domains, nuclear(TC2N)	RG	Homo sapiens
ENSG00000165949	interferon alpha inducible protein 27(IFI27)	RG	Homo sapiens
ENSG00000166145	serine peptidase inhibitor, Kunitz type 1(SPINT1)	RG	Homo sapiens
ENSG00000166292	transmembrane protein 100(TMEM100)	RG	Homo sapiens
ENSG00000166342	neuropilin and tolloid like 1(NETO1)	RG	Homo sapiens
ENSG00000166396	serpin family B member 7(SERPINB7)	RG	Homo sapiens
ENSG00000166670	matrix metallopeptidase 10(MMP10)	RG	Homo sapiens
ENSG00000166736	5-hydroxytryptamine receptor 3A(HTR3A)	RG	Homo sapiens
ENSG00000167105	transmembrane protein 92(TMEM92)	RG	Homo sapiens
ENSG00000167244	insulin like growth factor 2(IGF2)	RG	Homo sapiens
ENSG00000167748	kallikrein 1(KLK1)	RG	Homo sapiens
ENSG00000167850	CD300c molecule(CD300C)	RG	Homo sapiens
ENSG00000168267	pancreas associated transcription factor 1a(PTF1A)	RG	Homo sapiens
ENSG00000168398	bradykinin receptor B2(BDKRB2)	RG	Homo sapiens
ENSG00000168542	collagen type III alpha 1 chain(COL3A1)	RG	Homo sapiens
ENSG00000168671	UDP glycosyltransferase family 3 member A2(UGT3A2)	RG	Homo sapiens
ENSG00000168824	neuronal vesicle trafficking associated 1(NSG1)	RG	Homo sapiens
ENSG00000168843	follistatin like 5(FSTL5)	RG	Homo sapiens
ENSG00000168961	galectin 9(LGALS9)	RG	Homo sapiens
ENSG00000169031	collagen type IV alpha 3 chain(COL4A3)	RG	Homo sapiens
ENSG00000169218	R-spondin 1(RSPO1)	RG	Homo sapiens
ENSG00000169429	C-X-C motif chemokine ligand 8(CXCL8)	RG	Homo sapiens
ENSG00000169676	dopamine receptor D5(DRD5)	RG	Homo sapiens
ENSG00000169860	purinergic receptor P2Y1(P2RY1)	RG	Homo sapiens
ENSG00000170099	serpin family A member 6(SERPINA6)	RG	Homo sapiens
ENSG00000170162	vestigial like family member 2(VGLL2)	RG	Homo sapiens
ENSG00000170214	adrenoceptor alpha 1B(ADRA1B)	RG	Homo sapiens
ENSG00000170356	olfactory receptor family 2 subfamily A member 20 pseudogene(OR2A20P)	RG	Homo sapiens
ENSG00000170381	semaphorin 3E(SEMA3E)	RG	Homo sapiens
ENSG00000170430	O-6-methylguanine-DNA methyltransferase(MGMT)	RG	Homo sapiens
ENSG00000170454	keratin 75(KRT75)	RG	Homo sapiens
ENSG00000170500	LON peptidase N-terminal domain and ring finger 2(LONRF2)	RG	Homo sapiens
ENSG00000170579	DLG associated protein 1(DLGAP1)	RG	Homo sapiens
ENSG00000170689	homeobox B9(HOXB9)	RG	Homo sapiens
ENSG00000170745	potassium voltage-gated channel modifier subfamily S member 3(KCNS3)	RG	Homo sapiens
ENSG00000170786	short chain dehydrogenase/reductase family 16 C member 5(SDR16C5)	RG	Homo sapiens
ENSG00000170891	cytokine like 1(CYTL1)	RG	Homo sapiens
ENSG00000171044	XK related 6(XKR6)	RG	Homo sapiens
ENSG00000171388	apelin(APLN)	RG	Homo sapiens
ENSG00000171405	X antigen family member 5(XAGE5)	RG	Homo sapiens
ENSG00000171522	prostaglandin E receptor 4(PTGER4)	RG	Homo sapiens
ENSG00000171551	endothelin converting enzyme like 1(ECEL1)	RG	Homo sapiens
ENSG00000171714	anoctamin 5(ANO5)	RG	Homo sapiens
ENSG00000171714	uncharacterized LOC102723370(LOC102723370)	RG	Homo sapiens
ENSG00000171766	glycine amidinotransferase(GATM)	RG	Homo sapiens
ENSG00000172020	growth associated protein 43(GAP43)	RG	Homo sapiens
ENSG00000172031	epoxide hydrolase 4(EPHX4)	RG	Homo sapiens
ENSG00000172243	C-type lectin domain containing 7A(CLEC7A)	RG	Homo sapiens
ENSG00000172554	syntrophin gamma 2(SNTG2)	RG	Homo sapiens
ENSG00000172817	cytochrome P450 family 7 subfamily B member 1(CYP7B1)	RG	Homo sapiens
ENSG00000172995	cAMP regulated phosphoprotein 21(ARPP21)	RG	Homo sapiens
ENSG00000173068	basonuclin 2(BNC2)	RG	Homo sapiens
ENSG00000173208	ATP binding cassette subfamily D member 2(ABCD2)	RG	Homo sapiens
ENSG00000173705	sushi domain containing 5(SUSD5)	RG	Homo sapiens
ENSG00000173805	huntingtin associated protein 1(HAP1)	RG	Homo sapiens
ENSG00000173917	homeobox B2(HOXB2)	RG	Homo sapiens
ENSG00000174059	CD34 molecule(CD34)	RG	Homo sapiens
ENSG00000174567	golgi transport 1A(GOLT1A)	RG	Homo sapiens
ENSG00000174607	UDP glycosyltransferase 8(UGT8)	RG	Homo sapiens
ENSG00000175832	ETS variant transcription factor 4(ETV4)	RG	Homo sapiens
ENSG00000175928	leucine rich repeat neuronal 1(LRRN1)	RG	Homo sapiens
ENSG00000176658	myosin ID(MYO1D)	RG	Homo sapiens
ENSG00000176746	MAGE family member B6(MAGEB6)	RG	Homo sapiens
ENSG00000176907	transcriptional and immune response regulator(TCIM)	RG	Homo sapiens
ENSG00000177272	potassium voltage-gated channel subfamily A member 3(KCNA3)	RG	Homo sapiens
ENSG00000177519	reprimo, TP53 dependent G2 arrest mediator homolog(RPRM)	RG	Homo sapiens
ENSG00000178075	GRAM domain containing 1C(GRAMD1C)	RG	Homo sapiens
ENSG00000178163	zinc finger protein 518B(ZNF518B)	RG	Homo sapiens
ENSG00000178789	CD300 molecule like family member b(CD300LB)	RG	Homo sapiens
ENSG00000179023	kelch domain containing 7A(KLHDC7A)	RG	Homo sapiens
ENSG00000179046	tripartite motif family like 2(TRIML2)	RG	Homo sapiens
ENSG00000179399	glypican 5(GPC5)	RG	Homo sapiens
ENSG00000179542	SLIT and NTRK like family member 4(SLITRK4)	RG	Homo sapiens
ENSG00000179776	cadherin 5(CDH5)	RG	Homo sapiens
ENSG00000180613	GS homeobox 2(GSX2)	RG	Homo sapiens
ENSG00000181449	SRY-box transcription factor 2(SOX2)	RG	Homo sapiens
ENSG00000181541	mab-21 like 2(MAB21L2)	RG	Homo sapiens
ENSG00000181585	transmembrane inner ear(TMIE)	RG	Homo sapiens
ENSG00000182578	colony stimulating factor 1 receptor(CSF1R)	RG	Homo sapiens
ENSG00000182674	potassium voltage-gated channel subfamily B member 2(KCNB2)	RG	Homo sapiens
ENSG00000182742	homeobox B4(HOXB4)	RG	Homo sapiens
ENSG00000182747	solute carrier family 35 member D3(SLC35D3)	RG	Homo sapiens
ENSG00000182836	phosphatidylinositol specific phospholipase C X domain containing 3(PLCXD3)	RG	Homo sapiens
ENSG00000182916	transcription elongation factor A like 7(TCEAL7)	RG	Homo sapiens
ENSG00000183098	glypican 6(GPC6)	RG	Homo sapiens
ENSG00000183251	olfactory receptor family 51 subfamily B member 4(OR51B4)	RG	Homo sapiens
ENSG00000183421	receptor interacting serine/threonine kinase 4(RIPK4)	RG	Homo sapiens
ENSG00000183876	arylsulfatase family member I(ARSI)	RG	Homo sapiens
ENSG00000184254	aldehyde dehydrogenase 1 family member A3(ALDH1A3)	RG	Homo sapiens
ENSG00000184305	coiled-coil serine rich protein 1(CCSER1)	RG	Homo sapiens
ENSG00000184307	zinc finger DHHC-type palmitoyltransferase 23(ZDHHC23)	RG	Homo sapiens
ENSG00000184374	collectin subfamily member 10(COLEC10)	RG	Homo sapiens
ENSG00000184515	brain expressed X-linked 5(BEX5)	RG	Homo sapiens
ENSG00000184731	family with sequence similarity 110 member C(FAM110C)	RG	Homo sapiens
ENSG00000184905	transcription elongation factor A like 2(TCEAL2)	RG	Homo sapiens
ENSG00000184937	WT1 transcription factor(WT1)	RG	Homo sapiens
ENSG00000185149	neuropeptide Y receptor Y2(NPY2R)	RG	Homo sapiens
ENSG00000185272	RNA binding motif protein 11(RBM11)	RG	Homo sapiens
ENSG00000185352	heparan sulfate 6-O-sulfotransferase 3(HS6ST3)	RG	Homo sapiens
ENSG00000185652	neurotrophin 3(NTF3)	RG	Homo sapiens
ENSG00000185885	interferon induced transmembrane protein 1(IFITM1)	RG	Homo sapiens
ENSG00000185933	calcium homeostasis modulator 1(CALHM1)	RG	Homo sapiens
ENSG00000186197	EDAR associated via death domain(EDARADD)	RG	Homo sapiens
ENSG00000186838	selenoprotein V(SELENOV)	RG	Homo sapiens
ENSG00000187094	cholecystokinin(CCK)	RG	Homo sapiens
ENSG00000187821	helt bHLH transcription factor(HELT)	RG	Homo sapiens
ENSG00000187848	purinergic receptor P2X 2(P2RX2)	RG	Homo sapiens
ENSG00000188511	MIR3667 host gene(MIR3667HG)	RG	Homo sapiens
ENSG00000188620	H6 family homeobox 3(HMX3)	RG	Homo sapiens
ENSG00000188816	H6 family homeobox 2(HMX2)	RG	Homo sapiens
ENSG00000188883	killer cell lectin like receptor G2(KLRG2)	RG	Homo sapiens
ENSG00000189348	family with sequence similarity 90 member A27, pseudogene(FAM90A27P)	RG	Homo sapiens
ENSG00000196104	SPARC (osteonectin), cwcv and kazal like domains proteoglycan 3(SPOCK3)	RG	Homo sapiens
ENSG00000196126	major histocompatibility complex, class II, DR beta 1(HLA-DRB1)	RG	Homo sapiens
ENSG00000196352	CD55 molecule (Cromer blood group)(CD55)	RG	Homo sapiens
ENSG00000196482	estrogen related receptor gamma(ESRRG)	RG	Homo sapiens
ENSG00000196549	membrane metalloendopeptidase(MME)	RG	Homo sapiens
ENSG00000196569	laminin subunit alpha 2(LAMA2)	RG	Homo sapiens
ENSG00000196684	hematopoietic SH2 domain containing(HSH2D)	RG	Homo sapiens
ENSG00000196730	death associated protein kinase 1(DAPK1)	RG	Homo sapiens
ENSG00000197106	solute carrier family 6 member 17(SLC6A17)	RG	Homo sapiens
ENSG00000197249	serpin family A member 1(SERPINA1)	RG	Homo sapiens
ENSG00000197565	collagen type IV alpha 6 chain(COL4A6)	RG	Homo sapiens
ENSG00000197594	ectonucleotide pyrophosphatase/phosphodiesterase 1(ENPP1)	RG	Homo sapiens
ENSG00000198010	DLG associated protein 2(DLGAP2)	RG	Homo sapiens
ENSG00000198478	SH3 domain binding glutamate rich protein like 2(SH3BGRL2)	RG	Homo sapiens
ENSG00000198483	ankyrin repeat domain 35(ANKRD35)	RG	Homo sapiens
ENSG00000198515	cyclic nucleotide gated channel subunit alpha 1(CNGA1)	RG	Homo sapiens
ENSG00000198626	ryanodine receptor 2(RYR2)	RG	Homo sapiens
ENSG00000198680	tumor suppressor candidate 1(TUSC1)	RG	Homo sapiens
ENSG00000198848	carboxylesterase 1(CES1)	RG	Homo sapiens
ENSG00000203727	sterile alpha motif domain containing 5(SAMD5)	RG	Homo sapiens
ENSG00000203837	pancreatic lipase related protein 3(PNLIPRP3)	RG	Homo sapiens
ENSG00000203877	ripply transcriptional repressor 2(RIPPLY2)	RG	Homo sapiens
ENSG00000203900	uncharacterized LOC100130587(LOC100130587)	RG	Homo sapiens
ENSG00000204161	transmembrane protein 273(TMEM273)	RG	Homo sapiens
ENSG00000204287	major histocompatibility complex, class II, DR alpha(HLA-DRA)	RG	Homo sapiens
ENSG00000204291	collagen type XV alpha 1 chain(COL15A1)	RG	Homo sapiens
ENSG00000204442	NALCN channel auxiliary factor 1(NALF1)	RG	Homo sapiens
ENSG00000204603	long intergenic non-protein coding RNA 1257(LINC01257)	RG	Homo sapiens
ENSG00000204941	pregnancy specific beta-1-glycoprotein 5(PSG5)	RG	Homo sapiens
ENSG00000205221	vitrin(VIT)	RG	Homo sapiens
ENSG00000206432	transmembrane protein 200C(TMEM200C)	RG	Homo sapiens
ENSG00000211892	immunoglobulin heavy constant gamma 4 (G4m marker)(IGHG4)	RG	Homo sapiens
ENSG00000213214	Rho guanine nucleotide exchange factor 35(ARHGEF35)	RG	Homo sapiens
ENSG00000213937	claudin 9(CLDN9)	RG	Homo sapiens
ENSG00000214049	urothelial cancer associated 1(UCA1)	RG	Homo sapiens
ENSG00000214513	notochord homeobox(NOTO)	RG	Homo sapiens
ENSG00000214548	maternally expressed 3(MEG3)	RG	Homo sapiens
ENSG00000214866	doublecortin domain containing 2C(DCDC2C)	RG	Homo sapiens
ENSG00000215218	ubiquitin conjugating enzyme E2 Q family like 1(UBE2QL1)	RG	Homo sapiens
ENSG00000215612	H6 family homeobox 1(HMX1)	RG	Homo sapiens
ENSG00000218819	tudor domain containing 15(TDRD15)	RG	Homo sapiens
ENSG00000219438	TAFA chemokine like family member 5(TAFA5)	RG	Homo sapiens
ENSG00000224982	transmembrane protein 233(TMEM233)	RG	Homo sapiens
ENSG00000228705	long intergenic non-protein coding RNA 659(LINC00659)	RG	Homo sapiens
ENSG00000229621	long intergenic non-protein coding RNA 1822(LINC01822)	RG	Homo sapiens
ENSG00000230316	FEZF1 antisense RNA 1(FEZF1-AS1)	RG	Homo sapiens
ENSG00000230461	PROX1 antisense RNA 1(PROX1-AS1)	RG	Homo sapiens
ENSG00000231389	major histocompatibility complex, class II, DP alpha 1(HLA-DPA1)	RG	Homo sapiens
ENSG00000231924	pregnancy specific beta-1-glycoprotein 1(PSG1)	RG	Homo sapiens
ENSG00000234602	multiciliate differentiation and DNA synthesis associated cell cycle protein(MCIDAS)	RG	Homo sapiens
ENSG00000234841	TATA-box binding protein associated factor 4b pseudogene(LOC100422627)	RG	Homo sapiens
ENSG00000235026	DPP10 antisense RNA 1(DPP10-AS1)	RG	Homo sapiens
ENSG00000235621	long intergenic non-protein coding RNA 494(LINC00494)	RG	Homo sapiens
ENSG00000235875	ARHGEF7 antisense RNA 2(ARHGEF7-AS2)	RG	Homo sapiens
ENSG00000236507	uncharacterized LOC107987032(LOC107987032)	RG	Homo sapiens
ENSG00000237330	ring finger protein 223(RNF223)	RG	Homo sapiens
ENSG00000241157	ribosomal protein L32 pseudogene 32(RPL32P32)	RG	Homo sapiens
ENSG00000249242	transmembrane protein 150C(TMEM150C)	RG	Homo sapiens
ENSG00000253230	MIR124-1 host gene(MIR124-1HG)	RG	Homo sapiens
ENSG00000253301	long intergenic non-protein coding RNA 1606(LINC01606)	RG	Homo sapiens
ENSG00000254535	poly(A) binding protein cytoplasmic 4 like(PABPC4L)	RG	Homo sapiens
ENSG00000255624	C10orf88B (pseudogene)(C10orf88B)	RG	Homo sapiens
ENSG00000257864	IQ motif containing F1 pseudogene(LOC100294713)	RG	Homo sapiens
ENSG00000258597	serpin family A member 2 (gene/pseudogene)(SERPINA2)	RG	Homo sapiens
ENSG00000258991	double homeobox 4 like 19 (pseudogene)(DUX4L19)	RG	Homo sapiens
ENSG00000259029	double homeobox 4 like 18 (pseudogene)(DUX4L18)	RG	Homo sapiens
ENSG00000259070	long intergenic non-protein coding RNA 639(LINC00639)	RG	Homo sapiens
ENSG00000259527	long intergenic non-protein coding RNA 52(LINC00052)	RG	Homo sapiens
ENSG00000259881	uncharacterized LOC101927793(LOC101927793)	RG	Homo sapiens
ENSG00000260027	homeobox B7(HOXB7)	RG	Homo sapiens
ENSG00000266265	KLF transcription factor 14(KLF14)	RG	Homo sapiens
ENSG00000268388	FOXF1 adjacent non-coding developmental regulatory RNA(FENDRR)	RG	Homo sapiens
ENSG00000270164	long intergenic non-protein coding RNA 1480(LINC01480)	RG	Homo sapiens
ENSG00000272398	CD24 molecule(CD24)	RG	Homo sapiens
ENSG00000273079	glutamate ionotropic receptor NMDA type subunit 2B(GRIN2B)	RG	Homo sapiens
ENSG00000275620	uncharacterized LOC100192386(FLJ16779)	RG	Homo sapiens
ENSG00000277268	LHX1 divergent transcript(LHX1-DT)	RG	Homo sapiens
ENSG00000277632	C–C motif chemokine ligand 3(CCL3)	RG	Homo sapiens
ENSG00000278530	charged multivesicular body protein 1B2, pseudogene(CHMP1B2P)	RG	Homo sapiens
ENSG00000285722	uncharacterized LOC124905143(LOC124905143)	RG	Homo sapiens
ENSG00000286042	lung cancer associated lncRNA 1(LCAL1)	RG	Homo sapiens
ENSG00000287382	uncharacterized LOC105377663(LOC105377663)	RG	Homo sapiens
ENSG00000288638	lncRNA divergent activator of TBXT(LNCDAT)	RG	Homo sapiens
ENSG00000289070	uncharacterized LOC107985074(LOC107985074)	RG	Homo sapiens
ENSG00000289281	Vegetative cell wall protein gp1-like (A0A8I5KYS1_HUMAN)	RG	Homo sapiens

### Gene enrichment and KEGG pathway analysis

We submitted 601 overexpressed genes to functional annotation and KEGG pathway analysis using DAVID Bioinformatics Resources (https://david.ncifcrf.gov/home.jsp). Only pathways significantly enriched with –log10(p-value) less than 0.01 were considered. Both Bonferroni and Benjamini corrections were applied to limit the false detection rate (FDR) during analysis.

### Brain bulk tissue gene expression analysis of 33 enriched genes in neuroactive ligand-receptor interaction

The bulk tissue gene expression profiling was performed using GTEx Analysis Release V8 (dbGaP Accession phs000424.v8.p2) (https://www.gtexportal.org/). The Genotype-Tissue Expression (GTEx) initiative is building a public archive for studying tissue-specific gene expression and regulation. GTEX considers a population of 1000 persons sampled from 54 disease-free tissue sites for computing predictions. The main reason for collecting these samples was to do molecular assays like WGS, WES, and RNA-seq. Additional samples are available from GTEx Biobank. Gene expression, QTLs, and histology images are freely available on the GTEx Portal.

### Collection of genes with a high mutation frequency in GBM

Genes associated with GBM and showing high mutation frequency were obtained from COSMIC Database (https://cancer.sanger.ac.uk/cosmic) using the cancer browser. Top-20 genes that show high mutation frequency for the tissue/histology selections (Frontal Lobe) (*n* = 565) of the central nervous system (*n* = 17500) were identified. There was a total of 16 entries related to mutations in genes from 548 samples of the Glioblastoma. Data related to mutational matrix showing correlation with the gene expression profiles was also obtained from the COSMIC Database.

### Identification of Protein–Protein Interaction (PPI) connectivities using STRING

STRING database was used to predict the physical association and interaction among the 33 genes with other relevant proteins that may assist in the development of glioblastoma. STRING database (https://string-db.org/) is a resource rich website containing gene information, analyze gene lists and prioritize genes for physical and functional interactions with a high accuracy of prediction algorithm. We used it to show the interactions of the queried genes, i.e., 33 overexpressed genes implicated in neuroactive ligand receptor interaction, the pathway which showed high enrichment score during KEGG pathway analysis. All the query proteins were subject to PPI network prediction using the official gene symbol and resultant PPI networks were obtained at medium confidence of 0.400 in full STRING network type.

### Mann–Whitney analysis

Mann–Whitney analysis was performed to connect all somatic mutation status to gene expression changes in brain lower grade glioma. A box-plot analysis (differential analysis) of the CCK expression associated with mutation status of a gene was ascertained using the analysis. Similarly, to identify genes showing differential expression based on mutation status of IDH1 gene was also ascertained using the analysis. The p-value cut off was set to < 0.01 with a fold change (FC) cut-off set at 1.44.

### Prognostic overall survival analysis

The Kaplan–Meier based prognostic analysis of overall survival was plotted. The graphs were generated from the survival data from CPTAC by using R package "survival", "ggplot2", and "survminer" and splitting patients by upper 50%.

## Results

### More than 600 genes are differentially overexpressed in glioblastoma

**Fig. 1:**
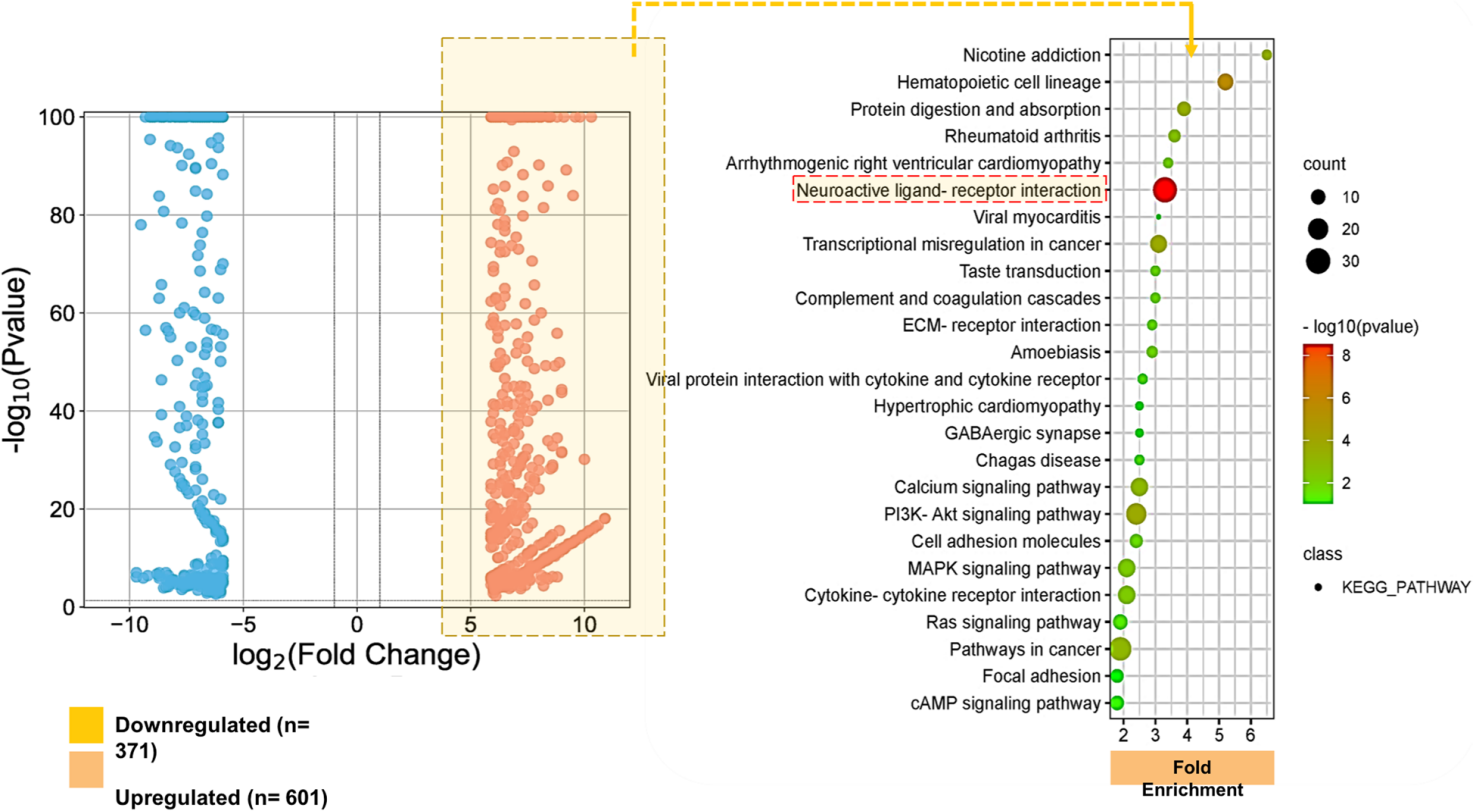
A volcano plot representation of 972 differentially regulated genes (upregulated *n* = 601; downregulated *n* = 301) (left panel). The fold enrichment analysis of the differentially upregulated genes showing different pathways involved in cancers (right panel)

Genes differentially overexpressed in GBM were retrieved from the public database (https://www.ebi.ac.uk/gxa/) (Accessed on July 28, 2023; Time: 14:30PM IST). We found a total of 972 genes differentially expressed in GBM based on experimental evidences from Microarray 1-colour and RNA-seq differential gene expression data. From the list of the genes upregulated and downregulated, only genes upregulated (based on their log_2_-fold change) were downloaded and saved into the local computer for further investigation (Fig. 1). We found 601 differentially upregulated genes in GBM and were submitted for further downstream analyses.

### Genes related to neuroactive ligand receptor interaction were enriched in KEGG pathway analysis

Out of 601 differentially upregulated genes, only 527 genes were read and annotated by DAVID Bioinformatics Resources (https://david.ncifcrf.gov/home.jsp) (Table 2). A total of 229 (43.5%) of genes were enriched in KEGG pathway. Out of 229 genes, 33 genes were (14.41%) predicted to be specifically and significantly enriched in the Neuroactive Ligand Receptor Interaction with a fold enrichment of 3.3 and –log10 p value of 3.00E-09. All 33 genes have been listed in Table 3.

**Table 2:** KEGG Pathway analysis of genes upregulated in Glioblastoma

Category	class	count	-log10(pvalue)	Fold Enrichment	Bonferroni	Benjamini	FDR
KEGG_PATHWAY	Focal adhesion	10	9.70E-02	1.8	1.00E + 00	8.60E-01	8.40E-01
KEGG_PATHWAY	Hypertrophic cardiomyopathy	6	9.50E-02	2.5	1.00E + 00	8.60E-01	8.40E-01
KEGG_PATHWAY	GABAergic synapse	6	9.20E-02	2.5	1.00E + 00	8.60E-01	8.40E-01
KEGG_PATHWAY	cAMP signaling pathway	11	8.20E-02	1.8	1.00E + 00	8.60E-01	8.40E-01
KEGG_PATHWAY	Viral myocarditis	5	7.80E-02	3.1	1.00E + 00	8.60E-01	8.40E-01
KEGG_PATHWAY	Chagas disease	7	5.70E-02	2.5	1.00E + 00	7.10E-01	6.90E-01
KEGG_PATHWAY	Ras signaling pathway	12	5.40E-02	1.9	1.00E + 00	7.10E-01	6.90E-01
KEGG_PATHWAY	Viral protein interaction with cytokine and cytokine receptor	7	5.30E-02	2.6	1.00E + 00	7.10E-01	6.90E-01
KEGG_PATHWAY	ECM-receptor interaction	7	3.30E-02	2.9	1.00E + 00	4.80E-01	4.70E-01
KEGG_PATHWAY	Taste transduction	7	2.80E-02	3	1.00E + 00	4.40E-01	4.30E-01
KEGG_PATHWAY	Complement and coagulation cascades	7	2.80E-02	3	1.00E + 00	4.40E-01	4.30E-01
KEGG_PATHWAY	Cell adhesion molecules	10	2.60E-02	2.4	1.00E + 00	4.40E-01	4.30E-01
KEGG_PATHWAY	Amoebiasis	8	2.00E-02	2.9	9.90E-01	3.80E-01	3.70E-01
KEGG_PATHWAY	Arrhythmogenic right ventricular cardiomyopathy	7	1.70E-02	3.4	9.90E-01	3.60E-01	3.50E-01
KEGG_PATHWAY	MAPK signaling pathway	17	7.50E-03	2.1	8.50E-01	1.70E-01	1.70E-01
KEGG_PATHWAY	Cytokine-cytokine receptor interaction	17	6.40E-03	2.1	8.00E-01	1.60E-01	1.60E-01
KEGG_PATHWAY	Rheumatoid arthritis	9	3.50E-03	3.6	5.80E-01	9.70E-02	9.40E-02
KEGG_PATHWAY	Calcium signaling pathway	17	1.30E-03	2.5	2.80E-01	4.10E-02	4.00E-02
KEGG_PATHWAY	Pathways in cancer	28	1.00E-03	1.9	2.20E-01	3.60E-02	3.50E-02
KEGG_PATHWAY	Nicotine addiction	7	6.40E-04	6.5	1.50E-01	2.60E-02	2.60E-02
KEGG_PATHWAY	Protein digestion and absorption	11	4.30E-04	3.9	1.00E-01	2.20E-02	2.10E-02
KEGG_PATHWAY	Transcriptional misregulation in cancer	16	2.10E-04	3.1	5.20E-02	1.30E-02	1.30E-02
KEGG_PATHWAY	PI3K-Akt signaling pathway	23	2.10E-04	2.4	5.10E-02	1.30E-02	1.30E-02
KEGG_PATHWAY	Hematopoietic cell lineage	14	2.20E-06	5.2	5.50E-04	2.80E-04	2.70E-04
KEGG_PATHWAY	Neuroactive ligand-receptor interaction	33	3.00E-09	3.3	7.50E-07	7.50E-07	7.40E-07

### Bulk tissue gene expression profiling revealed significant expression of CCK in brain cortex, frontal cortex- BA9 and BA24

The gene expression profile of bulk tissue was conducted using GTEx Analysis Release V8, which can be accessed through the dbGaP Accession phs000424.v8.p2. The GTEx Analysis Release V8 can be found at https://www.gtexportal.org/. The Genotype-Tissue Expression (GTEx) project is an ongoing initiative with the objective of creating a comprehensive and publically accessible archive for studying the expression and control of genes specific to different tissues. A sample collection was conducted, comprising 54 tissue regions that exhibited no signs of illness. The sample population consisted of around 1000 individuals. The main objective of the collection of these samples was to carry out molecular analyses, specifically Whole Genome Sequencing (WGS), Whole Exome Sequencing (WES), and RNA-Sequencing (RNA-Seq). Additional samples can be acquired through the GTEx Biobank. The GTEx Portal provides unlimited access to a diverse array of data, encompassing gene expression, quantitative trait loci (QTLs), and histology images.

**Table 3:** Description of 33 genes having involvement in neuroactive ligand-receptor interaction

ENSEMBL_GENE_ID	Symbol	Name
ENSG00000150556	LYPD6B	LY6/PLAUR domain containing 6B(LYPD6B)
ENSG00000128564	VGF	VGF nerve growth factor inducible(VGF)
ENSG00000170214	ADRA1B	adrenoceptor alpha 1B(ADRA1B)
ENSG00000128165	ADM2	adrenomedullin 2(ADM2)
ENSG00000171388	APLN	apelin(APLN)
ENSG00000100739	BDKRB1	bradykinin receptor B1(BDKRB1)
ENSG00000168398	BDKRB2	bradykinin receptor B2(BDKRB2)
ENSG00000110680	CALCA	calcitonin related polypeptide alpha(CALCA)
ENSG00000118432	CNR1	cannabinoid receptor 1(CNR1)
ENSG00000187094	CCK	cholecystokinin(CCK)
ENSG00000101204	CHRNA4	cholinergic receptor nicotinic alpha 4 subunit(CHRNA4)
ENSG00000164220	F2RL2	coagulation factor II thrombin receptor like 2(F2RL2)
ENSG00000125730	C3	complement C3(C3)
ENSG00000169676	DRD5	dopamine receptor D5(DRD5)
ENSG00000069482	GAL	galanin and GMAP prepropeptide(GAL)
ENSG00000151834	GABRA2	gamma-aminobutyric acid type A receptor subunit alpha2(GABRA2)
ENSG00000102287	GABRE	gamma-aminobutyric acid type A receptor subunit epsilon(GABRE)
ENSG00000163285	GABRG1	gamma-aminobutyric acid type A receptor subunit gamma1(GABRG1)
ENSG00000126010	GRPR	gastrin releasing peptide receptor(GRPR)
ENSG00000065325	GLP2R	glucagon like peptide 2 receptor(GLP2R)
ENSG00000152578	GRIA4	glutamate ionotropic receptor AMPA type subunit 4(GRIA4)
ENSG00000273079	GRIN2B	glutamate ionotropic receptor NMDA type subunit 2B(GRIN2B)
ENSG00000113749	HRH2	histamine receptor H2(HRH2)
ENSG00000132911	NMUR2	neuromedin U receptor 2(NMUR2)
ENSG00000164128	NPY1R	neuropeptide Y receptor Y1(NPY1R)
ENSG00000185149	NPY2R	neuropeptide Y receptor Y2(NPY2R)
ENSG00000101188	NTSR1	neurotensin receptor 1(NTSR1)
ENSG00000101327	PDYN	prodynorphin(PDYN)
ENSG00000125384	PTGER2	prostaglandin E receptor 2(PTGER2)
ENSG00000171522	PTGER4	prostaglandin E receptor 4(PTGER4)
ENSG00000187848	P2RX2	purinergic receptor P2X 2(P2RX2)
ENSG00000169860	P2RY1	purinergic receptor P2Y1(P2RY1)
ENSG00000146469	VIP	vasoactive intestinal peptide(VIP)

**Fig. 2:**
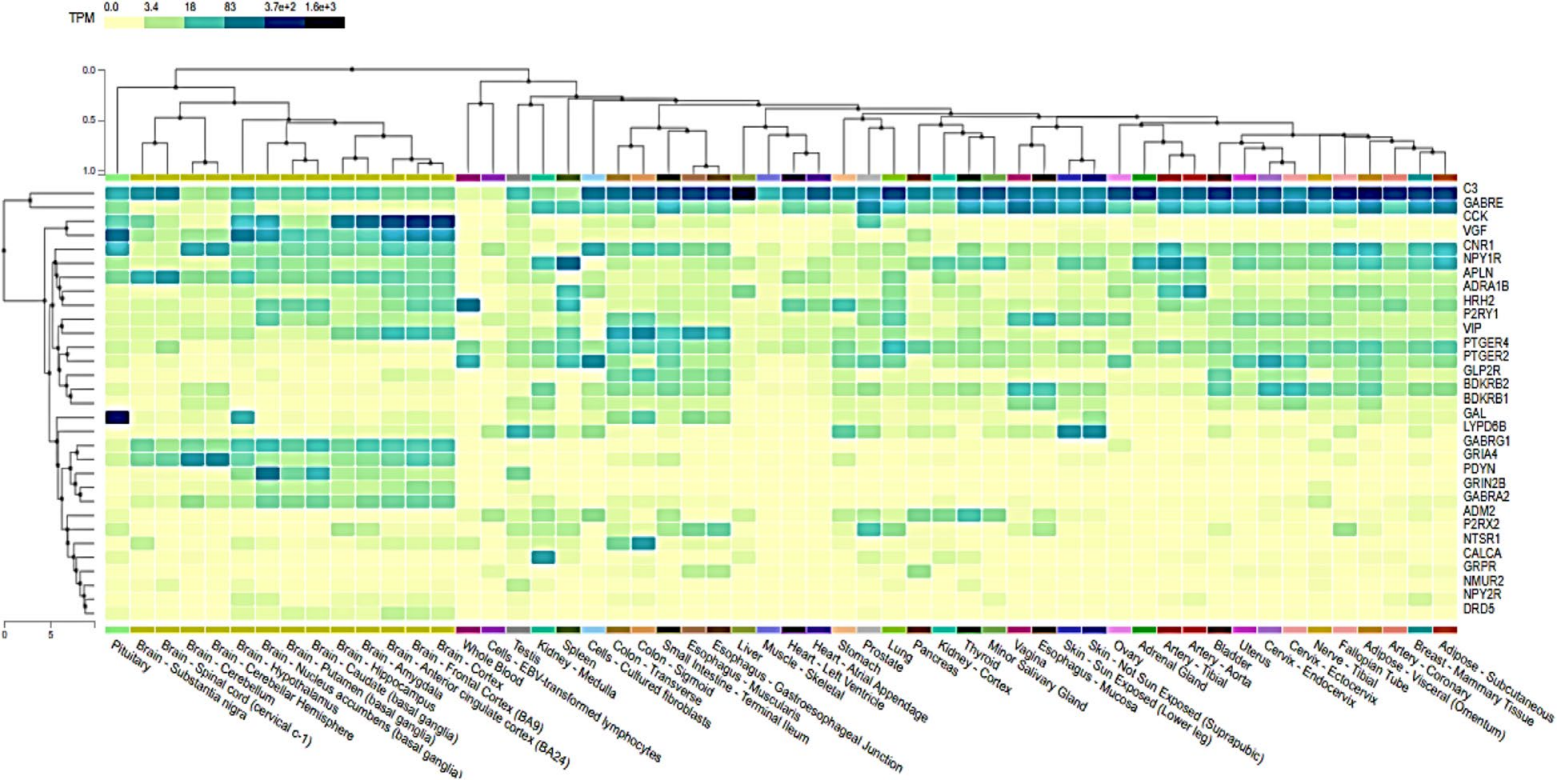
Brain bulk tissue gene expression analysis of 33 enriched genes in neuroactive ligand-receptor interaction (represented in the form of heatmaps) in different parts of brain

**Fig. 3:**
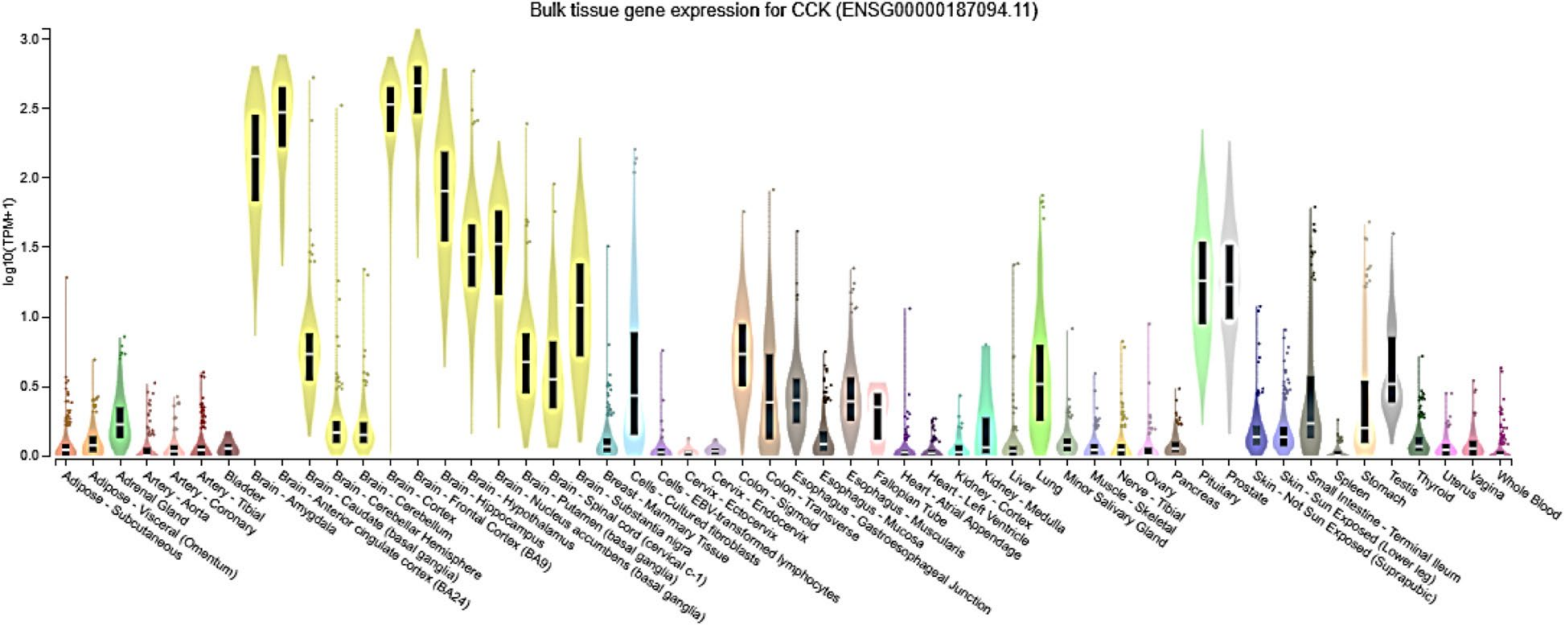
Brain bulk tissue gene expression analysis of 33 enriched genes in neuroactive ligand-receptor interaction (represented in the form of bar) in different parts of brain

**Fig. 4:**
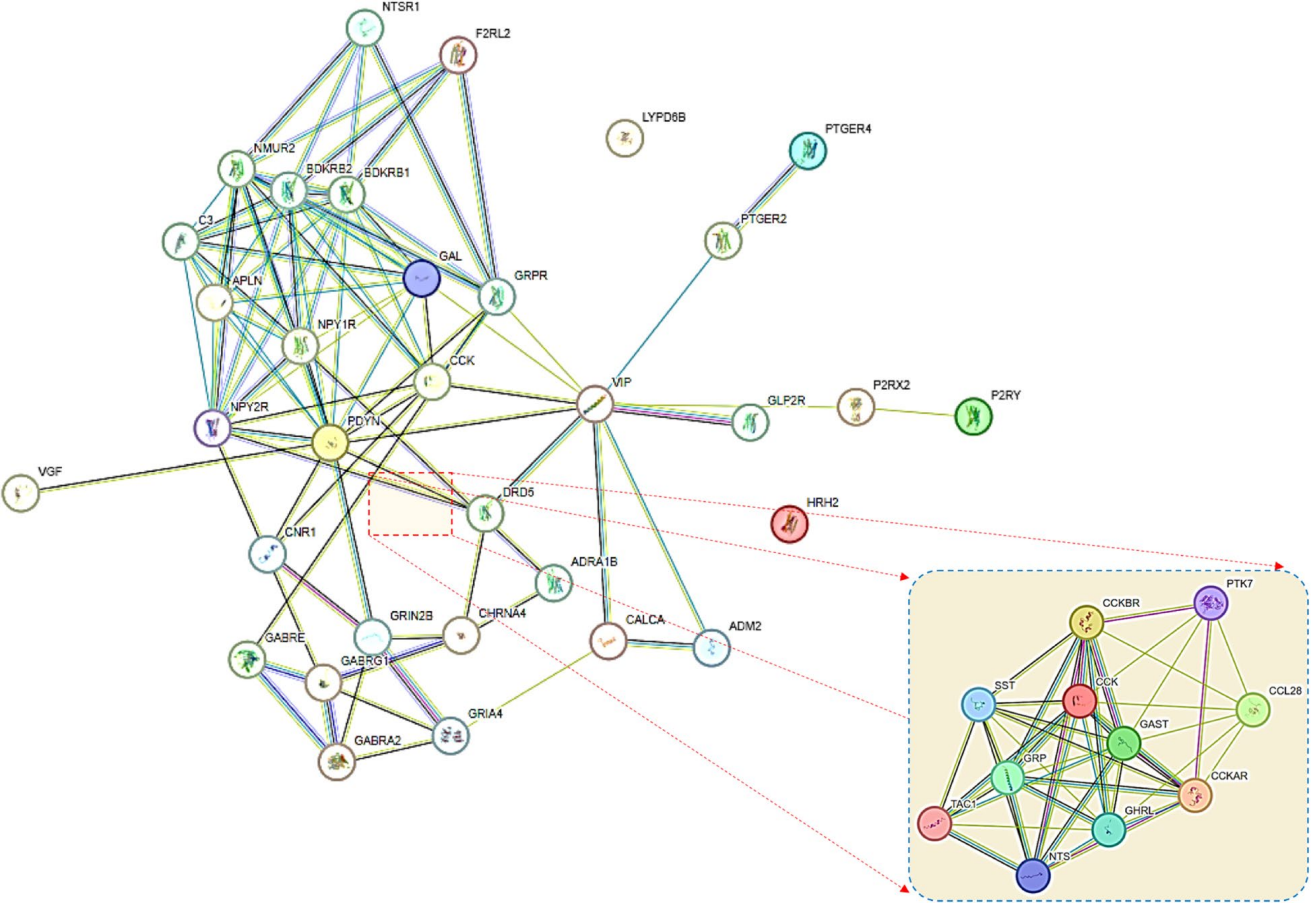
String-based network analysis of the 33 enriched genes in neuroactive ligand-receptor interaction

The bulk tissue gene expression profiling of 33 enriched genes revealed significantly high expression of CCK in cortex, frontal cortex- BA9 and BA24 region of the brain (Figs. 2 and 3). There was also moderate expression of CCK in hippocampus and amygdala (Fig. 2). The VGF expression was moderate in cortex, frontal cortex- BA9, BA24 region, basal ganglia and hypothalamus of the brain (Fig. 2). Expression of APLN was moderately detectable in substantia nigra and spinal cord (Fig. 2). CNR1 and GRIA4 showed moderate expression levels in cerebellar hemisphere and cerebellum (Fig. 2). PDYN expression was moderate in basal ganglia (Fig. 2). The expression profiles of other 27 genes were very low and considered insignificant for further analyses (Fig. 2). Similarly, PPI network analyses showed that while all 27 proteins have physical and functional interactions with each other, CCK mainly shows interactions with CCKBR, GAST, GRP, CCKAR, TAC1, NTS, GHRL and SST (Fig. 4). Although the current study did not find a significant prognostic difference between CCK-high/low or ZBTB20-high/low gliomas, the observed association between CCK expression and ZBTB20 mutation status suggests potential as a diagnostic or predictive biomarker. These findings may aid in stratifying patients and guiding therapeutic decisions, but further functional and clinical validation is necessary to establish their utility in clinical practice.

### IDH1, TERT, SETD2, BRAF and ATRX showed high mutation frequency in glioblastoma

**Fig. 5:**
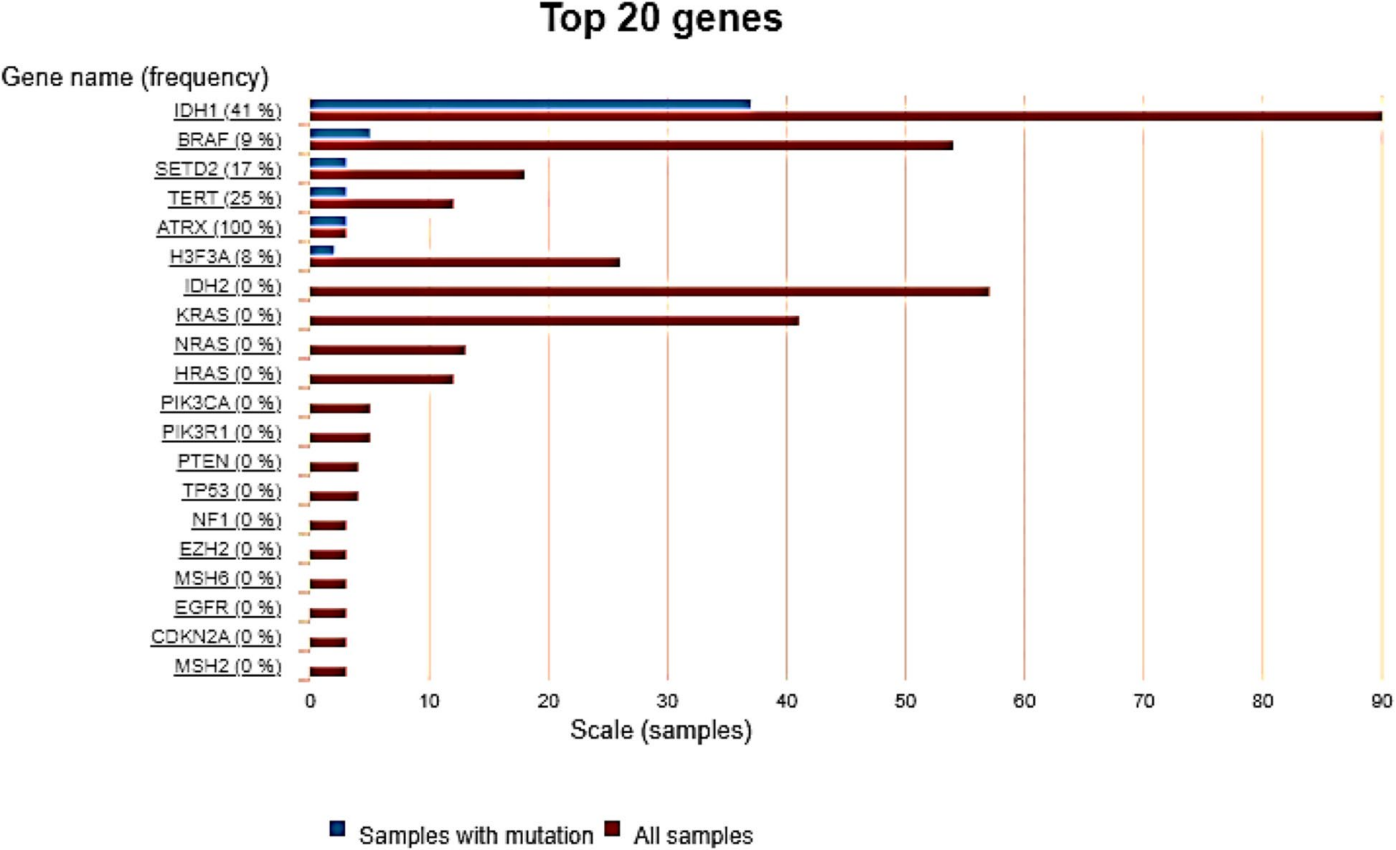
Mutation frequency of 33 enriched genes in neuroactive ligand-receptor interaction

Genes associated with PC and showing high mutation frequency were obtained from COSMIC Database (https://cancer.sanger.ac.uk/cosmic) using the cancer browser. Top-20 genes that show high mutation frequency for the tissue/histology selections (frontal lobe) (*n* = 565) of the central nervous system (*n* = 17500). There was a total of 16 entries related to mutations in genes from 548 samples of the Glioblastoma. Data related to mutational matrix showing correlation with the gene expression profiles was also obtained from the COSMIC Database. Mutational scoring of brain cortex and frontal cortex- BA9 revealed high prevalence and frequency of mutations in IDH1 (49%), TERT (25%), SETD2 (17%), BRAF (9%) (Fig. 5). The mutation frequency in ATRX was 100%; however, only computed using 3 studies, hence its role in GBM tumorigenesis cannot be attributed to highest mutational scoring. Based on results obtained, we could only corroborated indications of glioblastoma (GBM) malignancy and therapeutic responses to the overexpression of CCK and the presence of IDH1 mutations in tissue samples from brain cortex and frontal cortex.

**Fig. 6:**
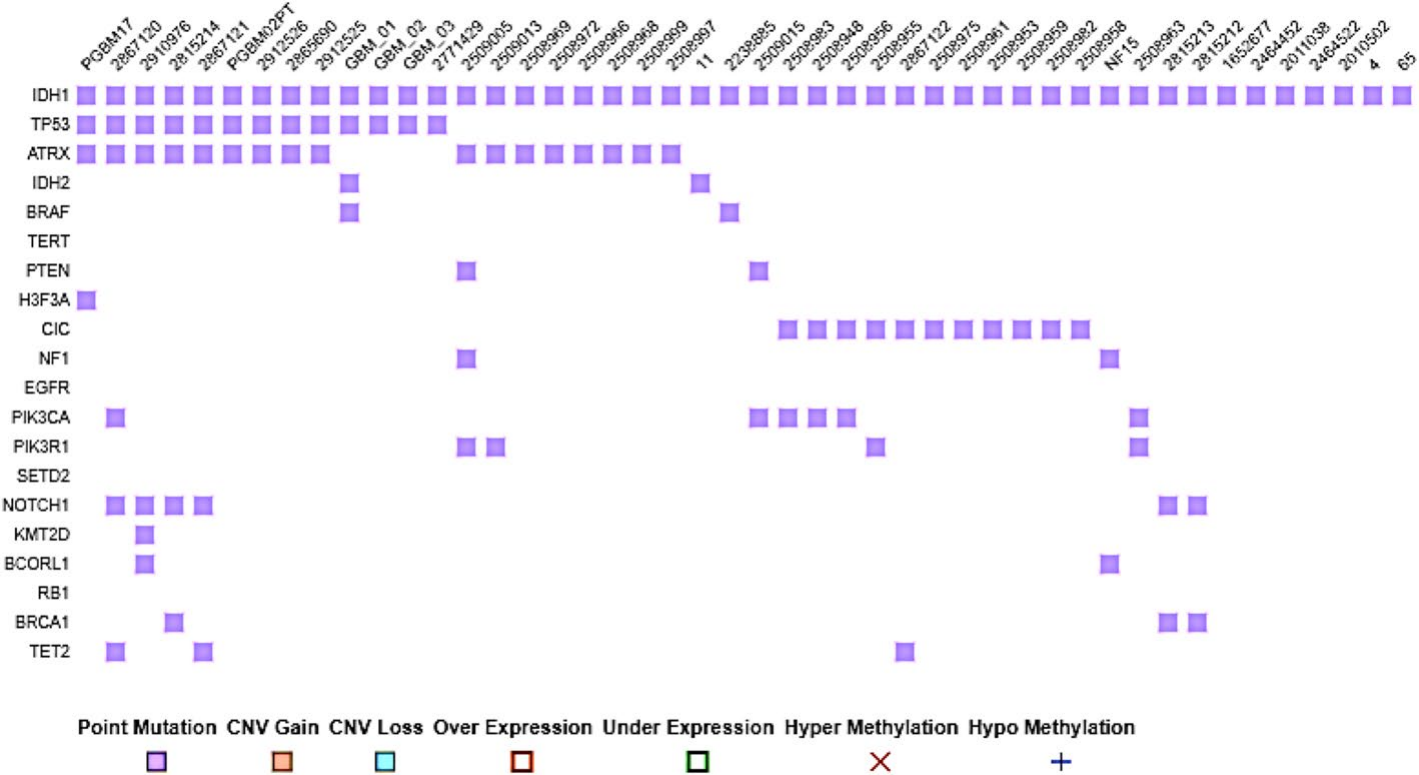
Different types of mutations in 33 enriched genes in neuroactive ligand-receptor interaction in different studies

The mutational matrix of the GBM using the datasets obtained from tissue selection of frontal lobe showed that in most of the GBM cases (41%), the IDH1 mutation is present (Fig. 5). Besides IDH1, TP53 and ATRX mutations were also scored in GBM samples. The data suggests that IDH1 alone or in combination with TP53 or ATRX or other gene mutations in varied combination cause GBM tumorigenesis (Fig. 6).

### CCK expression linked to zbtb20 mutations

In order to ascertain any possible association between CCK expression with somatic mutations in any gene, we performed Mann–Whitney analysis. The was performed to connect all somatic mutation status to gene expression changes in brain lower grade glioma. A box-plot analysis (differential analysis) of the CCK expression associated with mutation status of a gene was ascertained using the analysis. Similarly, to identify genes showing differential expression based on mutation status of IDH1 gene was also ascertained using the analysis. The p-value cut of was set to < 0.01 with a fold change (FC) cutoff set at 1.44 [7]. 

**Fig. 7:**
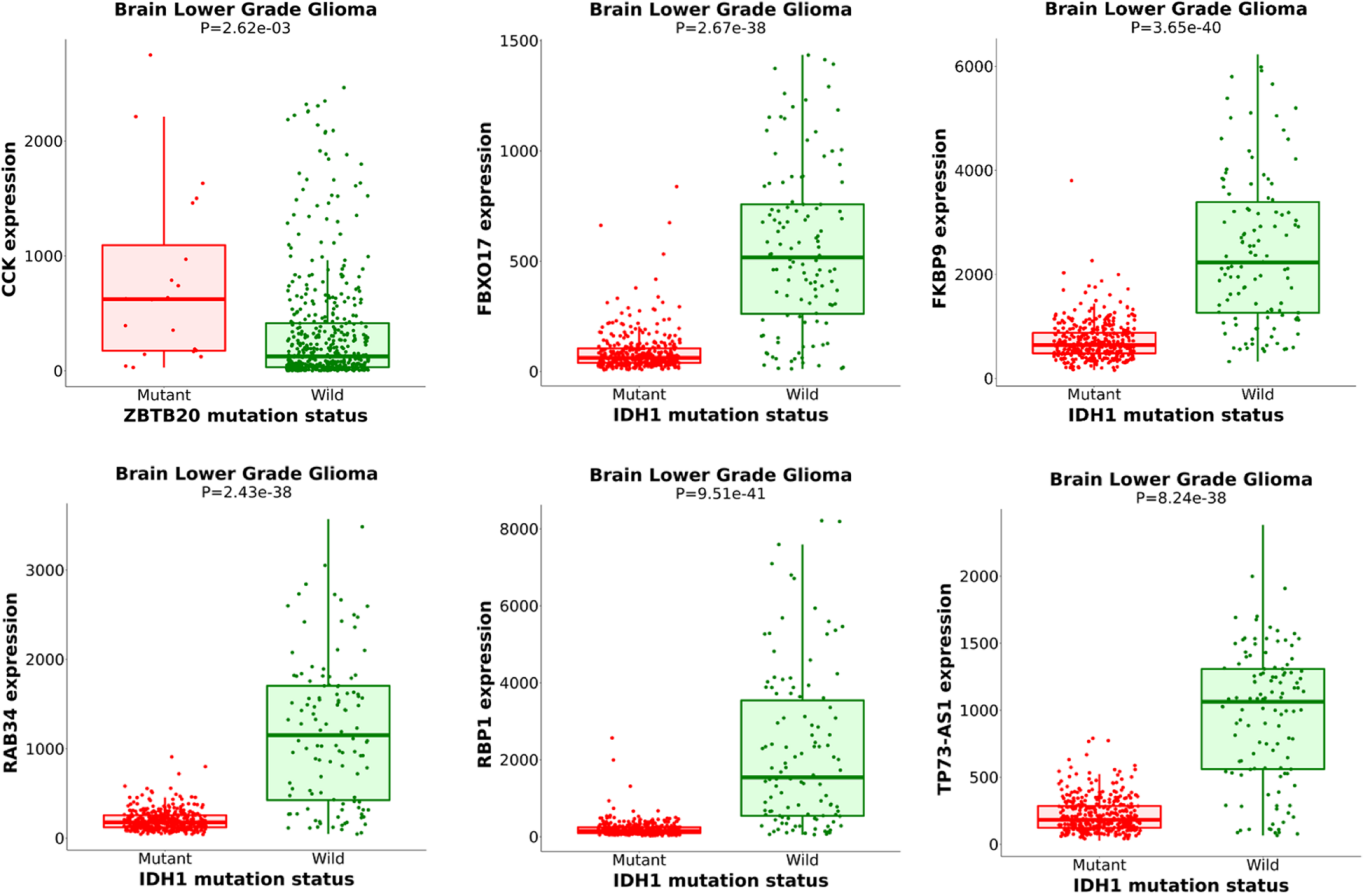
Correlation between expression of a gene and mutation status of another gene implicated in neuroactive ligand-receptor interaction

We found that CCK expression is linked to ZBTB20 mutation, wherein mutations cause overexpression of the CCK gene in glioblastoma (Fig. 7A). Whereas, CCK expression was not found to be linked to IDH1 mutations. Additionally, IDH1 mutations were predicted to cause changes in the expression of FBXO17, FKBP9, RAB34, RBP1, TP73-AS1 (Fig. 7B-F).

### CCK, IDH1 and ZBTB20 expression showed no prognostic relevance in glioblastoma

**Fig. 8:**
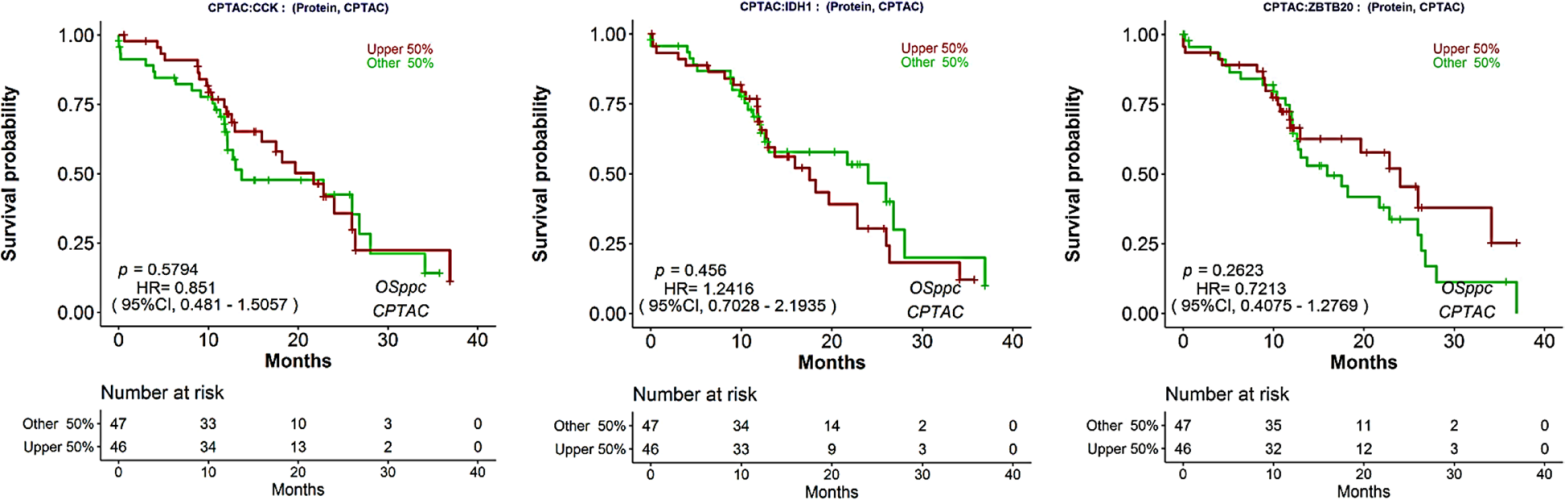
Survival probability of CCK, IDH1 and ZBTB20 in neuroactive ligand-receptor interaction

The Kaplan–Meier based prognostic analysis of overall survival was plotted for gene CCK, IDH1 and ZBTB20. The graphs were generated from the survival data from CPTAC by using R package "survival", "ggplot2", and "survminer" and splitting patients by upper 50% using OSpcc (https://bioinfo.henu.edu.cn/Protein/OSppc.html) [8]. The data showed that none of the test genes (CCK, IDH1 and ZBTB20) showed any prognostic relevance based on the overall survival (OS) analysis (Fig. 8).

## Discussion

In the process of disease diagnosis, it is crucial to analyse the genotypes of specific genes, namely isocitrate dehydrogenase (IDH), tumour protein p53 (TP53), and alpha thalassemia/mental retardation syndrome X linked (ATRX), as well as the presence of 1p/19q codeletion. Therefore, it is crucial to ascertain the presence of IDH mutation and 1p/19q status in order to classify dispersed gliomas, such as astrocytoma, oligoastrocytoma, oligodendroglioma, and glioblastoma, according to the 2016 classification [1]. The RAS/RAF/MEK/extracellular signal-regulated kinase (ERK) mitogen-activated protein kinase (MAPK) pathway plays a crucial role in transmitting mitogenic signals by activating growth factor receptors. This system is essential for various cellular processes including cell proliferation, survival, and differentiation. The importance of aberrant activation of RAS/RAF signalling in different types of tumours has been well-documented, and extensive research has demonstrated the significant clinical relevance of the MAPK pathway in gliomas [9]. The hyperactivation of the RAS/RAF pathway, leading to the development of different neoplasms, can be attributed to oncogenic mutations, copy number amplification of RAS/RAF, and aberrant activation of upstream growth factor receptors [10].

GBM is well recognised as the most aggressive tumour found within the central nervous system, exhibiting a median survival rate of 14–16 months. Despite the use of intensive multi-modal treatments, such as the combination of surgical resection, chemotherapy, and radiotherapy, the prognosis for patients with GBM remains unfavourable. Herein, we executed a systematic bioinformatics analysis on gene expression datasets from GXA and found thirty-three overexpressed genes in GBM that were enriched in neuroactive ligand receptor interaction. Subsequently, the bulk brain tissue gene expression profiling revealed high to moderate expression of CCK, VGF, APLN, CNR1, GRIA4 and PDYN in cortex, frontal cortex- BA9, BA24 region, cerebellar hemisphere, cerebellum, hippocampus, amygdala, basal ganglia, hypothalamus, substantia nigra and spinal cord of the brain. Interesting the expression of CCK was unimaginably high in the cortex, frontal cortex- BA9, and BA24 region of the brain. Mutational scoring of brain cortex and frontal lobe revealed high prevalence and frequency of mutations in IDH1 (49%), TERT (25%), SETD2 (17%), BRAF (9%). Based on results obtained, we firstly corroborated indications of glioblastoma (GBM) malignancy and therapeutic responses to the overexpression of CCK and the presence of IDH1 mutations in tissue samples from brain cortex and frontal cortex. However, Mann–Whitney analysis suggests CCK expression is linked to ZBTB20 mutations and not to IDH1 mutations. Moreover, the expression of CCK was not found to be associated with overall survival. Duan and co-authors (2024) and Oikonomou and co-authors (2008) reported that as compared to lower grade glioma, the expression of ZBTB20 in GBM tissues was lower and CCK abundance is high (PMID: 38881713, PMID: 18423848). Furthermore, ZBTB20 acts as activator of TET1/Fas/Caspase-3 pathway suggesting its role as a tumor suppressor protein (PMID: 38881713). Altogether, results suggest that CCK expression and ZBTB20 mutations may be employed in clinical interventions as potential predictive and diagnostic markers, respectively. The current study failed to observe a prognostic difference in CCK-high/low gliomas or ZBTB20 high/low gliomas. A key limitation of the current study is the lack of experimental validation to confirm the observed in silico association between CCK expression and ZBTB20 mutations in GBM and lower-grade gliomas. Establishing a clinical or mechanistic link would require further investigation through functional assays, such as knockdown or overexpression studies in relevant cell line models.

Shibahara and co-authors (2018) documented the occurrence of GBM in four individuals diagnosed with neurofibromatosis type 1 (NF1), who did not have mutations in IDH1, BRAF V600E, and TERT promoter genes [11]. Each patient presented with an intracranial tumour that was well-defined and localised, and subsequently had surgical removal of the tumour along with concurrent chemoradiotherapy. At the 5-year follow-up, three individuals remained alive. The author posited that it is imperative to differentiate these tumours, which exhibit distinctive pathological characteristics, from the conventional IDH wildtype GBM [11]. The present example had comparable characteristics as described earlier. To the best of our current understanding, this represents the initial documentation of a case involving GBM in a female patient with neurofibromatosis type 1 (NF1), whereby the absence of IDH1, BRAF V600E, and TERT promoter mutations was seen [11]. Furthermore, the tumour exhibited reduced aggressiveness and displayed a clearly demarcated border. The patient demonstrated survival at the 13-month follow-up period subsequent to undergoing the conventional therapy protocol [11]. The findings provided additional support for the notion that these patients may represent a distinct subgroup within the context of GBM, hence enhancing the potential for future investigation. Nevertheless, the results of our study were constrained by the utilisation of an incomplete sample.

The majority of research on BRAF V600E in gliomas mostly examines paediatric neoplasms, with a particular emphasis on gangliogliomas and pleomorphic xanthoastrocytomas [12, 13]. The inclusion of exclusively adult patients in this study allows for a focused examination of the impact of BRAF mutations on glioma in the adult population. Furthermore, it should be noted that BRAF mutations possess prognostic significance in addition to their diagnostic role [14]. The analysis of our data reveals that the male gender constituted the predominant proportion of patients within the BRAF mutation cohort, in contrast to the BRAF AMP group. The prevalence of GBM was shown to be greater in the cohort with BRAF mutations compared to the cohort with BRAF amplifications (BRAF AMP). Conversely, the BRAF AMP group exhibited a considerably higher number of individuals with mutations in IDH1/2, TP53, and ATRX. ATRX deletions/mutations have been found to be linked with various conventional molecular occurrences, such as mutations in IDH1 and TP53 [15, 16]. Somatic mutations affecting the TP53, ATRX, and IDH1/2 genes have been detected in adult low-grade gliomas [17]. While primary GBM exhibits a low prevalence of IDH1/2 mutations, these alterations are frequently observed in diffuse/anaplastic gliomas and subsequent GBM [18, 19]. ATRX mutations have been identified in adult diffuse gliomas and astrocytomas that also have mutations in both TP53 and IDH1/2 genes. The concurrent presence of TP53, IDH1/2, and ATRX mutations has been observed to promote the proliferation of a specific subset of adult diffuse astrocytomas [20]. The aforementioned studies collectively demonstrate a high frequency of co-occurrence between ATRX mutations and mutations in IDH1 and TP53. Furthermore, our examination of the string data indicates significant associations among the BRAF, IDH1, IDH2, TP53, and ATRX proteins, consistent with findings reported in prior research [20]. Furthermore, these findings indicate that BRAF exhibits direct interactions with TP53 and IDH1, while no such interactions were observed with ATRX.

## Conclusion

Clinical practice increasingly uses molecular genetic classification to diagnose, prognose, and treat cancer. This is because it provides prompt diagnostic information, prognostic insights throughout disease progression, and individualized therapy guidance. Genetic tests should be done alongside histology investigation for a clear diagnosis. However, molecular markers must be evaluated quickly and accurately to be used in diagnostic processes. The absence of this assessment makes it difficult to validate data and make timely decisions in treating central nervous system tumor patients, particularly in selecting the best treatment protocols. Unfortunately, the quest for a biomarker that can be detected by high-throughput, cost-effective, and simply implementable technologies for urgent, reliable, and economical CNS illness diagnosis remains elusive. However, certain treatments may be rational but cost more patients. In the present study, we identified a significant association between CCK neuropeptide expression and ZBTB20 mutation status in gliomas using in silico analysis. While this relationship may have potential diagnostic, predictive, or prognostic implications, its clinical and functional relevance remains to be confirmed through further experimental validation.

## Supplementary Information

Supplementary Material 1.

## Data Availability

No datasets were generated or analysed during the current study.

## Abbreviations

LGG: Low-grade gliomas
HGG: High-grade gliomas
WGS: Whole Genome Sequencing
WES: Whole Exome Sequencing

## References

[1] Louis DN, et al. The 2016 world health organization classification of tumors of the central nervous system: a summary. Acta Neuropathol. 2016;131(6):803–20. 27157931 10.1007/s00401-016-1545-1

[2] Comprehensive. Integrative genomic analysis of diffuse lower-grade gliomas. N Engl J Med. 2015;372(26):2481–98. 26061751 10.1056/NEJMoa1402121 PMC4530011

[3] Ceccarelli M, et al. Molecular profiling reveals biologically discrete subsets and pathways of progression in diffuse glioma. Cell. 2016;164(3):550–63. 26824661 10.1016/j.cell.2015.12.028 PMC4754110

[4] Eckel-Passow JE, et al. Glioma groups based on 1p/19q, IDH, and TERT promoter mutations in tumors. N Engl J Med. 2015;372(26):2499–508. 26061753 10.1056/NEJMoa1407279 PMC4489704

[5] Karsy M, et al. New molecular considerations for glioma: IDH, ATRX, BRAF, TERT, H3 K27M. Curr Neurol Neurosci Rep. 2017;17(2):19. 28271343 10.1007/s11910-017-0722-5

[6] Johnson BE, et al. Mutational analysis reveals the origin and therapy-driven evolution of recurrent glioma. Science. 2014;343(6167):189–93. 24336570 10.1126/science.1239947 PMC3998672

[7] Nagy Á, Győrffy B. Mutarget: a platform linking gene expression changes and mutation status in solid tumors. Int J Cancer. 2021;148(2):502–11. 32875562 10.1002/ijc.33283

[8] Connor AA, et al. Integration of genomic and transcriptional features in pancreatic cancer reveals increased cell cycle progression in metastases. Cancer Cell. 2019;35(2):267-282.e7. 30686769 10.1016/j.ccell.2018.12.010 PMC6398439

[9] Lyustikman Y, et al. Constitutive activation of Raf-1 induces glioma formation in mice. Neoplasia. 2008;10(5):501–10. 18472967 10.1593/neo.08206 PMC2373912

[10] Jeuken J, et al. RAS/RAF pathway activation in gliomas: the result of copy number gains rather than activating mutations. Acta Neuropathol. 2007;114(2):121–33. 17588166 10.1007/s00401-007-0239-0

[11] Shibahara I, et al. Glioblastoma in neurofibromatosis 1 patients without IDH1, BRAF V600E, and TERT promoter mutations. Brain Tumor Pathol. 2018;35(1):10–8. 29138945 10.1007/s10014-017-0302-z

[12] Henson JD, et al. DNA c-circles are specific and quantifiable markers of alternative-lengthening-of-telomeres activity. Nat Biotechnol. 2009;27(12):1181–5. 19935656 10.1038/nbt.1587

[13] Sievert AJ, et al. Duplication of 7q34 in pediatric low-grade astrocytomas detected by high-density single-nucleotide polymorphism-based genotype arrays results in a novel BRAF fusion gene. Brain Pathol. 2009;19(3):449–58. 19016743 10.1111/j.1750-3639.2008.00225.x PMC2850204

[14] Dahiya S, et al. BRAF(V600E) mutation is a negative prognosticator in pediatric ganglioglioma. Acta Neuropathol. 2013;125(6):901–10. 23609006 10.1007/s00401-013-1120-y

[15] Cai J, et al. ATRX, IDH1-R132H and Ki-67 immunohistochemistry as a classification scheme for astrocytic tumors. Oncoscience. 2016;3(7–8):258–65. 27713914 10.18632/oncoscience.317 PMC5043074

[16] Modrek AS, et al. Low-grade astrocytoma mutations in IDH1, P53, and ATRX cooperate to block differentiation of human neural stem cells via repression of SOX2. Cell Rep. 2017;21(5):1267–80. 29091765 10.1016/j.celrep.2017.10.009 PMC5687844

[17] Kannan K, et al. Whole-exome sequencing identifies ATRX mutation as a key molecular determinant in lower-grade glioma. Oncotarget. 2012;3(10):1194–203. 23104868 10.18632/oncotarget.689 PMC3717947

[18] Yan H, et al. IDH1 and IDH2 mutations in gliomas. N Engl J Med. 2009;360(8):765–73. 19228619 10.1056/NEJMoa0808710 PMC2820383

[19] Parsons DW, et al. An integrated genomic analysis of human glioblastoma multiforme. Science. 2008;321(5897):1807–12. 18772396 10.1126/science.1164382 PMC2820389

[20] Liu XY, et al. Frequent ATRX mutations and loss of expression in adult diffuse astrocytic tumors carrying IDH1/IDH2 and TP53 mutations. Acta Neuropathol. 2012;124(5):615–25. 22886134 10.1007/s00401-012-1031-3

